# Panamanian frog species host unique skin bacterial communities

**DOI:** 10.3389/fmicb.2015.01171

**Published:** 2015-10-27

**Authors:** Lisa K. Belden, Myra C. Hughey, Eria A. Rebollar, Thomas P. Umile, Stephen C. Loftus, Elizabeth A. Burzynski, Kevin P. C. Minbiole, Leanna L. House, Roderick V. Jensen, Matthew H. Becker, Jenifer B. Walke, Daniel Medina, Roberto Ibáñez, Reid N. Harris

**Affiliations:** ^1^Department of Biological Sciences, Virginia TechBlacksburg, VA, USA; ^2^Smithsonian Tropical Research InstituteBalboa, Ancón, Republic of Panamá; ^3^Department of Chemistry, Villanova UniversityVillanova, PA, USA; ^4^Department of Statistics, Virginia TechBlacksburg, VA, USA; ^5^Department of Biology, James Madison UniversityHarrisonburg, VA, USA

**Keywords:** amphibian, *Batrachochytrium dendrobatidis*, chytrid fungus, Kolmogorov-Smirnov measure, microbiome, microbiota, structure-function relationship

## Abstract

Vertebrates, including amphibians, host diverse symbiotic microbes that contribute to host disease resistance. Globally, and especially in montane tropical systems, many amphibian species are threatened by a chytrid fungus, *Batrachochytrium dendrobatidis* (Bd), that causes a lethal skin disease. Bd therefore may be a strong selective agent on the diversity and function of the microbial communities inhabiting amphibian skin. In Panamá, amphibian population declines and the spread of Bd have been tracked. In 2012, we completed a field survey in Panamá to examine frog skin microbiota in the context of Bd infection. We focused on three frog species and collected two skin swabs per frog from a total of 136 frogs across four sites that varied from west to east in the time since Bd arrival. One swab was used to assess bacterial community structure using 16S rRNA amplicon sequencing and to determine Bd infection status, and one was used to assess metabolite diversity, as the bacterial production of anti-fungal metabolites is an important disease resistance function. The skin microbiota of the three Panamanian frog species differed in OTU (operational taxonomic unit, ~bacterial species) community composition and metabolite profiles, although the pattern was less strong for the metabolites. Comparisons between frog skin bacterial communities from Panamá and the US suggest broad similarities at the phylum level, but key differences at lower taxonomic levels. In our field survey in Panamá, across all four sites, only 35 individuals (~26%) were Bd infected. There was no clustering of OTUs or metabolite profiles based on Bd infection status and no clear pattern of west-east changes in OTUs or metabolite profiles across the four sites. Overall, our field survey data suggest that different bacterial communities might be producing broadly similar sets of metabolites across frog hosts and sites. Community structure and function may not be as tightly coupled in these skin symbiont microbial systems as it is in many macro-systems.

## Introduction

All animals serve as hosts to symbiotic microorganisms that, along with their genetic contributions, constitute their microbiome. We have long understood that the microbes that reside in the gut are diverse and are important in helping digest food. More recently, we have begun to appreciate the incredible diversity of these symbiotic microbial communities and to recognize that they reside throughout the body (Cho and Blaser, 2012; Ursell et al., 2012). The composition of these microbial communities can strongly influence many facets of host health and disease resistance (Cho and Blaser, 2012; Fierer et al., 2012; McFall-Ngai et al., 2013). Our knowledge in this area is growing in large part because of advances in molecular microbiology that now allow us to study these complex microbial communities in much more detail. Culture-independent studies, while initially focused on revealing diversity, now clearly indicate complex interactions in these communities both among microbes and among microbes and their host (McFall-Ngai et al., 2013; Boon et al., 2014; Manor et al., 2014).

Recent work on amphibians provides an example of the role that the natural microbiota may have in preventing disease. *Batrachochytrium dendrobatidis* (Bd), a fungal pathogen first isolated and described in 1999 (Longcore et al., 1999), causes the potentially lethal skin disease, chytridiomycosis, in susceptible amphibians. Bd has been associated with many amphibian population declines in recent decades since it was first observed in dead and dying frogs from both Central America and Australia (Berger et al., 1998). The adaptive and innate immune systems, and in particular the production of antimicrobial peptides in amphibian skin, has an important role in preventing Bd infection in some host species (McMahon et al., 2014; Rollins-Smith et al., 2015). However, the skin of healthy amphibians is also host to a diverse resident bacterial community (McKenzie et al., 2012; Walke et al., 2014), and a number of these bacteria can inhibit Bd growth (Harris et al., 2006; Flechas et al., 2012; Bell et al., 2013; Becker et al., 2015b; Woodhams et al., 2015). Inhibition is likely due to bacterially-produced secondary metabolites inhibiting Bd zoospore colonization or development (Brucker et al., 2008a,b; Becker et al., 2009; Lam et al., 2011; Bell et al., 2013). In addition, experiments have demonstrated that a supplemented protective microbiota can reduce morbidity and mortality in some amphibians infected with Bd (Harris et al., 2009), and that reduction of the cutaneous microbial community can worsen disease outcomes (Becker and Harris, 2010). Several recent experimental studies have also linked the structure of the microbial communities on the skin with Bd exposure (Jani and Briggs, 2014; Becker et al., 2015a) and infection outcome (Becker et al., 2015a). These studies illustrate that Bd may affect bacterial skin communities both by causing differential mortality of bacterial species and by selecting for hosts with protective bacterial communities.

Panamá has historically had one of the most diverse assemblages of amphibians in the Neotropics (reviewed in Jaramillo et al., 2010) and is one of the places where Bd was first described (Berger et al., 1998). Since its initial discovery, Bd has been moving eastward across Panamá (Lips et al., 2006; Woodhams et al., 2008; Rebollar et al., 2014), and its movement there has been closely monitored. As it has spread, many Panamanian amphibian populations have been decimated by Bd; at one highland site, 25 of 63 named amphibian species (~40%) disappeared following the arrival of Bd (Crawford et al., 2010). However, not all amphibian species are susceptible to chytridiomycosis (Smith et al., 2009; Crawford et al., 2010; Kilburn et al., 2010), and even some populations of what were assumed to be Bd-susceptible species in Panamá seem to be persisting at some sites with the fungus (Hertz et al., 2012; Perez et al., 2014). Many hypotheses have been put forward to explain this variation, including differential immune responses among species and variation in disease dynamics due to abiotic factors, such as temperature and humidity, that differ across sites (Blaustein et al., 2011, 2012; Venesky et al., 2014).

To explore the potential role of the skin microbiota in disease resistance in free-living Panamanian frogs, we used a field survey of three frog species across four sites on a west-east gradient in Panamá, with Bd having arrived earlier (~2006) at the western site and later (~2011) at the eastern site (Woodhams et al., 2008; Rebollar et al., 2014). We first described the microbiota of each frog host species. We then conducted a broader scale comparison of the microbiota from Panamanian species with that from three frog species from the eastern US, which have a much longer history of coexistence with Bd (Ouellet et al., 2005). Finally, we used the Panamá field survey dataset and the prior knowledge of the distribution of Bd in Panamá to examine the potential link between the structure of bacterial skin symbiont communities and their function in Bd disease resistance. We hypothesized that if Bd is a strong selective force on these symbiont communities, we should see: (1) individuals currently infected with Bd having different microbial community structure (OTUs) and function (metabolite profiles) than non-infected individuals, (2) changes in the microbial communities in terms of alpha- and beta-diversity along this west-east gradient, and less variance in metabolite profiles at sites with a longer history of Bd exposure as selection for anti-fungal production has occurred for a longer period of time, and (3) a stronger correlation between OTU community structure and metabolite production at sites with a longer history of Bd exposure.

## Materials and methods

### Panamá field survey: sample collection

**Figure 1:**
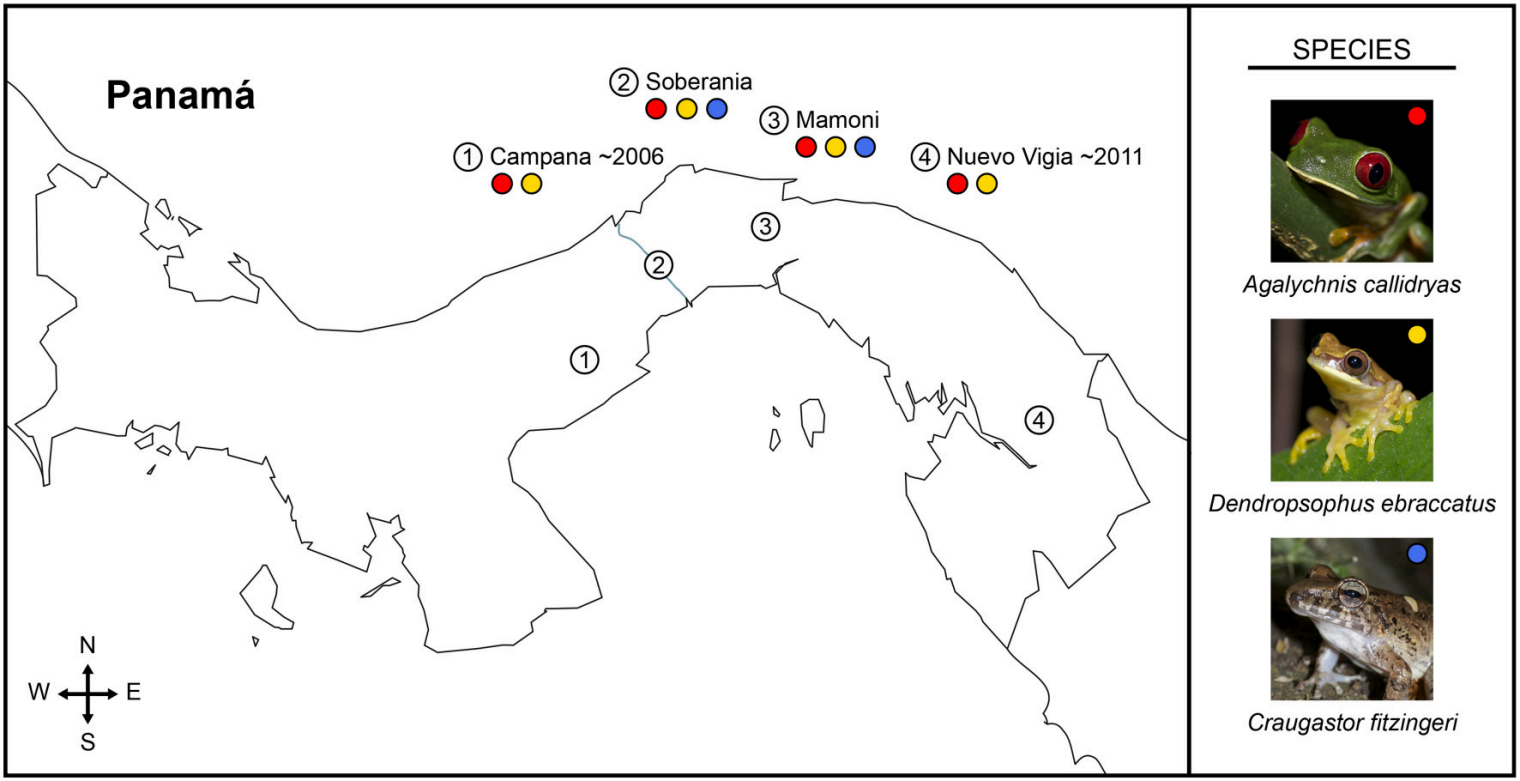
**Map of Panamá showing sampled field sites (numbered circles) and the frog species sampled at each location (colored circles: red, *Agalychnis callidryas*; gold, *Dendropsophus ebraccatus*; blue, *Craugastor fitzingeri*) during a field survey assessing the diversity of bacterial communities on amphibian skin**. The three species of amphibians we sampled have persisted with the fungal pathogen, *Batrachochytrium dendrobatidis*, for different lengths of time across the four sampled sites, numbered one to four from west to east (i.e., longest at Cerro Campana and shortest at Nuevo Vigia; Woodhams et al., 2008; Rebollar et al., 2014).

In 2012, we sampled 2 or 3 species of frogs at each of four sites in Panamá, for a total of 136 frogs (Table 1A). Study sites spanned from central to eastern Panamá (Figure 1). Sites, listed from west to east, were: Parque Nacional Altos de Campana (Panamá Province), Parque Nacional Soberanía (Panamá Province), the Mamoní Valley Preserve (Panamá Province), and forest surrounding the community of Nuevo Vigía (Darién Province). The elevation of the ponds and streams where frogs were encountered ranged from 29 to 824 m (Table 1A). All animal use was approved by the Institutional Animal Care and Use Committees of Virginia Tech and the Smithsonian Tropical Research Institute, and was completed with permission from the Autoridad Nacional del Ambiente in Panamá.

**Table 1:** **Summary of frog species, dates, and sites sampled during field surveys in Panamá (A) and the United States (B) assessing the diversity of bacterial communities on amphibian skin**.

**Species**	**Site, province (Panamá) or pond, county/city (US)**	**Elevation (m)**	**Sample size (A/J)**	**SVL (cm) mean, sd**	**Mass (g) mean, sd**
**(A) PANAMÁ**					
***Agalychnis callidryas***					
12 Sept 2012	Parque Nacional Altos de Campana, Panamá	824	15 (15/0)	4.7, 0.4	4.3, 1.9
29 Aug 2012	Parque Nacional Soberania, Panamá	50	15 (15/0)	4.8, 0.6	4.7, 2.7
9–11 July 2012	Mamoni Valley Preserve, Panamá	191	20 (20/0)	4.6, 0.5	4.1, 1.7
27 Sept 2012	Nuevo Vigia, Darien	29	12 (12/0)	4.6, 0.5	4.1, 1.6
***Craugastor fitzingeri***					
30 Aug and 6 Sept 2012	Parque Nacional Soberania, Panamá	64	14 (10/4)	3.5, 1.0	3.9, 2.6
12 July 2012	Mamoni Valley Preserve, Panamá	245	7 (4/3)	2.5, 0.3	1.1, 0.3
***Dendropsophus ebraccatus***					
12 Sept 2012	Parque Nacional Altos de Campana, Panamá	824	15(15/0)	2.9, 0.2	1.3, 0.4
29 Aug 2012	Parque Nacional Soberania, Panamá	50	14 (14/0)	2.6, 0.3	1.0, 0.5
9–11 July 2012	Mamoni Valley Preserve, Panamá	191	9 (9/0)	2.7, 0.1	1.0, 0.1
25 Sept 2012	Nuevo Vigia, Darien	29	15 (15/0)	2.9, 0.3	1.2, 0.4
**(B) UNITED STATES**					
***Anaxyrus americanus***					
20 March 2012	Craig Creek Road, Craig	526	9 (9/0)		
***Lithobates catesbeianus***					
4 April 2012	Station Pond, Giles	1176	9 (2/7)		
2 May 2012	Pandapas Pond, Montgomery	683	9 (3/6)		
2 April 2012	Peaks View Park, Lynchburg City	200	8 (5/3)		
1 April 2012	Reedy Creek, Richmond City	23	9 (0/9)		
***Pseudacris crucifer***					
4 April 2012	Sylvatica Pond, Giles	1181	9 (9/0)		
8 March 2012	Pandapas Pond, Montgomery	683	9 (9/0)		
2 April 2012	Food Lion, Campbell	269	7 (7/0)		
1 April 2012	James River Wetland, Richmond City	51	9 (9/0)		

Site elevation and sample characteristics are also summarized, as available, including sample size [total number of individuals as well as the number of adults (A), and juveniles (J)]; snout vent length in centimeters (SVL; mean and standard deviation of all individuals); and mass in grams (mean and standard deviation of all individuals).

Two species of treefrogs (Family: Hylidae), *Agalychnis callidryas* (red-eyed treefrogs) and *Dendropsophus ebraccatus* (pantless treefrogs), were sampled at all four sites (*A. callidryas*: *N* = 12–20 individuals/site; *D. ebraccatus*: *N* = 9–15 individuals/site; Table 1A). *Agalychnis callidryas* and *D. ebraccatus* are sympatric, breed in ponds and at each of the four sites were sampled at the same pond. One additional species, *Craugastor fitzingeri* (Fitzinger's robber frogs, Family: Craugastoridae), was only encountered, and thus sampled, at two of the four sites (*N* = 7 and 14 individuals/site; Table 1A). *Craugastor fitzingeri* was found along the margins of a stream at both sites where this species was sampled.

We sampled the amphibians' skin bacterial community diversity and metabolite profiles by swabbing the surface of the skin, and we determined if individuals were infected with the fungal pathogen, *B. dendrobatidis* (methods and individual Bd results are reported in Rebollar et al., 2014). To sample frogs, we captured them by hand, using new nitrile gloves for each individual. Individuals that could not be swabbed immediately were placed in sterile Whirl-Pak® bags (Nasco, Fort Atkinson, WI, USA) for a maximum of 30 min prior to swabbing. Before swabbing, we rinsed each frog by pouring ~50 ml of sterile deionized water over its body to remove any dirt and transient bacteria (Walke et al., 2014). Each individual was swabbed twice: first, to sample the cutaneous bacterial community and second, to sample metabolites. We sampled the cutaneous bacterial community using sterile rayon swabs (MW113, Medical Wire Equipment & Co. Ltd., Corsham, UK). We then used polyurethane-tipped swabs to assess metabolite profiles (14-960-3J, Fisher Scientific). Prior to use, all polyurethane swabs were pre-treated to remove methanol-soluble impurities by twice rinsing the swab in methanol and then allowing it to dry fully under sterile conditions in the laboratory (Umile et al., 2014). To standardize swabbing across individuals, we swabbed the ventral surface 20 times, each thigh 5 times, and each hind foot 5 times for a total of 40 strokes/swab type/individual. We recorded each individual's life stage (adult or juvenile), snout vent length, and mass before releasing the animal at the site of capture (summarized in Table 1A). We placed all swabs in sterile, empty 1.5 mL microcentrifuge tubes and stored them on ice or liquid nitrogen in the field. The swabs were then transferred them to a −80°C freezer in the laboratory until further processing.

### Panamá field survey: bacterial community structure

Relative abundance of OTUs based on 16S rRNA gene amplicon sequencing was used to assess bacterial community structure on the skin. DNA from the bacterial sampling swabs was extracted using the DNeasy Blood and Tissue Kit (Qiagen, Valencia CA, USA, Cat. 69506) according to manufacturer's protocol and including an initial lysozyme incubation step for 1 h at 37°C. DNA was eluted in a final volume of 100 μl, and was then used as template to detect Bd infection intensity and prevalence through qPCR as previously reported (Rebollar et al., 2014). In parallel, another portion of the DNA was used as a template for amplicon sequencing using barcoded primers targeting the V4 region of the 16S rRNA gene with primers 515F and 806R (Caporaso et al., 2012). The reverse PCR primer was barcoded with a 12-base error-correcting Golay code to allow for multiplexing of samples, and also contained sequencing adapter regions. PCR reactions contained 13 μL molecular grade PCR water, 10 μL 5 Prime Hot Master Mix, 0.5 μL each of the forward and reverse primers (10 μM final concentration), and 1.0 μL genomic DNA. PCR conditions were: denaturation step 3 min at 94°C, amplification step for 35 cycles for 45 s at 94°C, annealing for 60 s at 50°C, extension 90 s at 72°C, and a final extension of 10 min at 72°C. PCR was done in triplicate, pooled, checked for integrity in a 1% agarose gel and quantified in a QuantiFluor-ST using a florescent dye specific for dsDNA (Promega, Madison WI, USA, Cat. E6090 and Cat. E2670). Each set of 66 samples was pooled by adding 300 ng of DNA from each set of PCR products. Each pooled sample was then cleaned using the QIAquick PCR purification kit (Qiagen, Valencia CA, USA, Cat. 28104). The pooled samples were sent for 250PE Illumina amplicon sequencing at the Dana Farber Cancer Research Institute at Harvard University, Boston, MA.

Raw Illumina 16S rRNA amplicon data files were joined using Fastq-join v. 0.1 (Aronesty, 2011). Joined sequences (length restricted to 250–256 bp) were then processed and quality filtered using the QIIME pipeline (Caporaso et al., 2010b). We used the default settings for demultiplexing with the following exceptions: we allowed for no errors in the barcode, we increased the maximum number of consecutive low quality base calls allowed before truncating a read (r) to 10, and we decreased the minimum number of consecutive high quality base calls to include a read (p) to 0.5. Sequences were assigned to operational taxonomic units (OTUs) based on 97% sequence similarity using the UCLUST method (Edgar, 2010). To represent each OTU, we used the most abundant sequence from each cluster. Representative sequences were aligned to the Greengenes 13_5 reference database (DeSantis et al., 2006) using PyNAST (Caporaso et al., 2010a). Taxonomy was assigned using the RDP classifier (Wang et al., 2007). We removed OTUs with fewer than 0.001% of the total number of reads, which in this dataset was those with fewer than 112 reads (Bokulich et al., 2013). Sequencing depth per sample ranged from 7067 to 211,411 reads, so we rarefied all samples to a sequencing depth of 7000. Two samples for which there was no corresponding metabolite data were also excluded. The final dataset consisted of 3492 OTUs across 136 samples (62 from *A. callidryas*, 53 from *D. ebraccatus*, and 21 from *C. fitzingeri*). 16S rRNA amplicon sequences were deposited in the NCBI Sequence Read Archive (SRA study accession number: SRP062596).

### Panamá field survey: metabolite profiles

Metabolite profiles were considered a “fingerprint” of the metabolites present on the skin of each frog. Our methods focused on non-polar, small (< 600 m/z) metabolites that are likely to be produced by bacteria, as opposed to host compounds, such as anti-microbial peptides. We do not know which metabolites that comprise the profile are inhibitory to Bd, but we expect the presence of Bd to select for bacteria that produce metabolites that are inhibitory. For metabolite profile analysis, swabs were shipped frozen to Villanova University. To extract metabolites, 1.0 mL of HPLC-grade methanol was added to each swab in its tube. The tubes were capped and vortexed for 5 s, allowed to sit for 10 min, and then vortexed a second time. The swab tip was then removed using forceps. The methanolic extract was slowly filtered into another centrifuge tube using 13 mm syringe filters (0.2 μm PTFE membrane, VWR) to remove any insoluble environmental material. Before use, syringes (1 mL HSW Norm-Ject® disposable syringe) and filters were pre-washed by taking up 1 mL of methanol in the syringe and slowly passing it through the filter. Filtered extracts were evaporated *in vacuo* using a DNA120 SpeedVac with the heating function turned off.

Dried metabolite extracts were reconstituted in 100 μL of methanol containing 1 ppm naphthalene (as internal standard). The reconstituted extracts were analyzed by reversed-phase, high performance liquid chromatography (HPLC, 25 μL injection) using a Shimadzu LC-20 liquid chromatograph equipped with an ACE C18 column (3 μm, 150 × 4.6 mm), a Shimadzu SPD-M20A diode array detector, and an Applied Biosystems SCIEX API 2000 triple quadrupole mass spectrometer (operating in positive electrospray ionization mode). Compounds were separated with a binary mobile phase flowing at 0.5 mL min^−1^ consisting of acidified water (0.1% formic acid, *v/v*; Solvent A) and acidified acetonitrile (0.1% formic acid, *v/v*; Solvent B). The gradient was as follows: 10% B (2 min hold) ramped to a final mobile phase concentration of 100% B over 18 min (5 min hold). Total Wavelength Chromatograms (TWC) of field samples were compared against the TWC of extracted, unused and washed swabs (controls) and also blank, methanol injections. These methods best detect small hydrophobic molecules, such as alkaloids, and therefore do not capture larger and/or more polar molecules, such as antimicrobial peptides.

The retention times of all detected compounds (peaks) were normalized to that of the naphthalene internal standard (20.69 min). The retention time of each chromatographic feature was determined by manually integrating each peak in the TWC using Applied Biosystems Analyst software V.1.5.1. This data set was further manually revised to reduce its size by accounting for slight variations in retention time across multiple samples and focusing on major chemical components. First, compounds that eluted with retention times ± 0.03 min across all samples were investigated for UV-Vis chromophores (λ_max_) and positively-charged ions. Those compounds with both similar retention times and identical spectroscopic features were pooled and assigned as a single compound. Next, unique compounds only detected in a single sample were disregarded as noise. Finally, all features that had a peak area of less than 3000 mAU × min were disregarded as minor components.

### Comparison of the diversity of skin microbial communities of amphibians from panamá and the US

We compared the diversity of the skin bacterial communities of the three species from Panamá to three species from the US that co-occur with Bd: *Lithobates catesbeianus* (American bullfrogs, Family: Ranidae)*, Pseudacris crucifer* (spring peepers, Family: Hylidae), and *Anaxyrus americanus* (American toads, Family: Bufonidae). *L. catesbeianus* occupies aquatic habitat and typically breeds in permanent water bodies. Both *P. crucifer* and *A. americanus* occupy terrestrial habitat as adults but breed in a variety of aquatic habitats ranging from permanent to ephemeral. The three US species were sampled between March-May 2012 at several ponds in Virginia, USA, using the same methods as the present study (Table 1B; Walke et al., 2015; Belden et al., unpublished data). From Panamá, we included all four populations of *A. callidryas* and *D. ebraccatus*, as well as the two populations of *C. fitzingeri*. From the US, we included four populations of *L. catesbeianus* and *P. crucifer*, and one population of *A. americanus*. To standardize sample sizes, if more than nine individuals were sampled, we randomly chose nine individuals from each population to include in the analysis. Three populations had fewer than nine individuals: one *C. fitzingeri* population (*N* = 7), one *P. crucifer* population (*N* = 7), and one *L. catesbeianus* population (*N* = 8).

Demultiplexed sequences for all individuals from Panamá and the US were combined into a single.fasta file for OTU assignment, taxonomy assignment, and quality filtering. We used the same methods as above, except, for this dataset, we (a) removed OTUs with fewer than 289 reads based on the recommended 0.001% cutoff (Bokulich et al., 2013), and (b) all samples were rarefied to a sequencing depth of 10,000 sequences/sample.

### Statistical analysis

#### Overview of statistical approach

We first completed a general descriptive analysis of our field survey data. This consisted of general summaries of OTU and metabolite diversity of the three host species we studied. In addition, as we found species level differences in OTUs, we assessed what OTUs were responsible for those differences using a novel analysis, the Kolmogorov-Smirnov (K-S) Measure. We also compared alpha- and beta-diversity of bacterial communities from the three Panamanian frog species with samples from three frog species in Virginia, USA, which have likely had a much longer history with Bd (Ouellet et al., 2005). As there were clear differences between frogs from the two regions, we identified some of the key OTUs driving those differences. Then, we used our field survey dataset to assess our specific hypotheses regarding the structure (OTUs) and function (metabolites) of the skin communities on individuals with and without Bd and across the west-east gradient of sites in Panamá that varied in the length of time since Bd arrival. All analyses were run in R (R Core Team, 2014).

#### OTU and metabolite diversity on amphibians from panamá

We tested for differences in alpha-diversity of OTUs across species using richness, estimated as the number of unique OTUs/individual, and Faith's phylogenetic diversity. We evaluated community structure in terms of dominance/evenness using the Simpson index (Haegeman et al., 2013). The Simpson index varies between 0 and 1, with values closer to 0 indicating a more even community and values closer to 1 indicating a community dominated by fewer species. We also tested for differences in metabolite richness, estimated as the number of unique metabolites/individual, across species. OTU richness, OTU phylogenetic diversity, and metabolite richness were fit using linear mixed models (package lme4, Bates et al., 2014). The Simpson Index was fit using mixed effect beta regression models (package glmmADMB; Fournier et al., 2012; Skaug et al., 2014). For linear and generalized linear mixed models, we included “site” as a random effect to account for nestedness of samples from the same site. We report “adjusted means” for alpha-diversity measures in the results section that account for the random effects. To estimate P values of factors (e.g., species), we used likelihood ratio tests to compare nested models of increasing simplicity (Zuur et al., 2009). If analyses indicated significant differences among species, we conducted pairwise comparisons to determine which species were similar or different from one another. For linear mixed models, we used Tukey's Honest Significant Different tests to correct for multiple comparisons (function ghlt, package multcomp; Hothorn et al., 2008). This option was not available for the mixed effect beta regression models. Therefore, to determine which species were similar or different from one another, we reran the model on subsets of the dataset representing each pairwise species comparison. For analyses here and below, OTU richness and phylogenetic diversity were log-transformed to better meet assumptions of normality.

For beta-diversity, we tested whether variation in OTU community structure and metabolite profiles could be explained by species using permutational multivariate analysis of variance (PERMANOVA, Anderson, 2001) using the function adonis in the vegan package (Oksanen et al., 2013). Separate tests were run for OTUs (relative abundance) and metabolite profiles (presence/absence), and in each case we accounted for the sampling site in the models using the “strata” argument in the adonis function in R. Metabolite profiles were assessed based on presence/absence because the nature of our HPLC-MS method did not allow us to determine relative abundance of the different metabolites on individual frogs. Community data were transformed to distance matrices based on Bray–Curtis and Jaccard dissimilarities for the OTU and metabolite datasets, respectively. If species effects were significant, we conducted pairwise comparisons using PERMANOVA to determine which species were different from one another. To visualize these results, we used nonmetric multi-dimensional scaling (NMDS).

**Figure 2:**
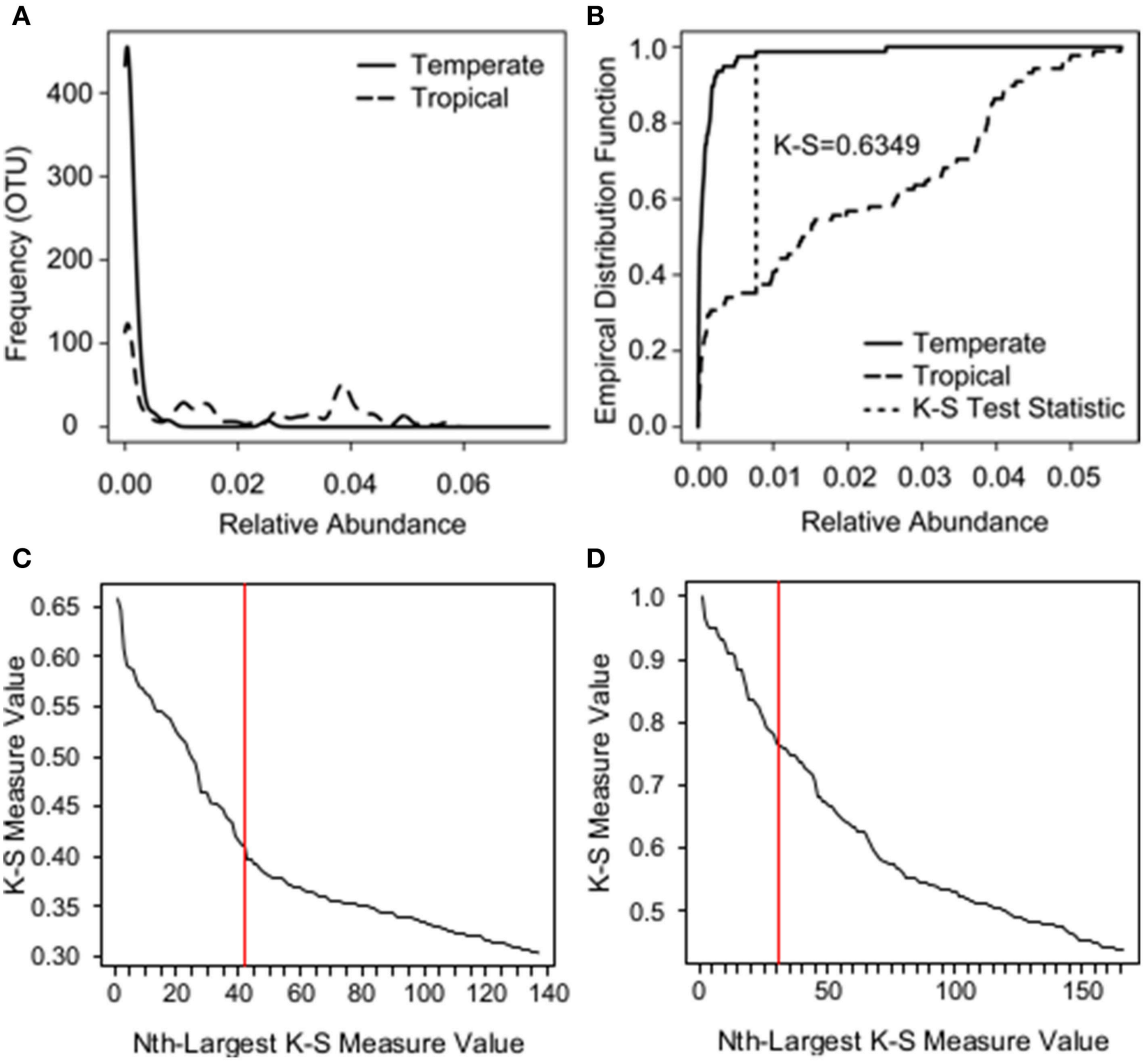
**Example of how the K-S test statistic is determined and K-S measures plotted in descending order for species and zone analyses**. **(A)** The relative abundance distributions of a single bacterial OTU across two groups: temperate amphibians (solid line) vs. tropical amphibians (long dash). **(B)** The K-S test statistic assesses the difference between the two distributions based on the largest absolute distance between empirical distribution functions (short dash). The empirical distribution function is an observed cumulative distribution function, where, in this case, the y-axis represents the proportion of observations less than or equal to a given relative abundance on the x-axis. **(C)** K-S measures for each OTU plotted in descending order for species analysis. **(D)** K-S measures for each OTU plotted in descending order for zone analysis.

To identify the OTUs driving the variation among the three frog species (*K* = 3), we used a variable screening technique called the K-S measure (Loftus et al., 2015). As discussed in Loftus et al. (2015), other approaches are available to isolate important OTUs, including Sure Information Screening (Fan and Lv, 2008), LASSO (Tibshirani, 1996), and Indicator Species Analyses (Da Caceres et al., 2010), however such methods rely on strong analytical assumptions and/or do not apply directly to a multinomial response. Thus, to relax analytical assumptions and assess the relationship between OTUs and frog species (a multinomial response), we use the K-S measure. The K-S measure is an extension of the K-S test statistic (Kolmogorov, 1933; Smirnov, 1936). The original K-S test statistic assesses the difference (or lack thereof) in *K* = 2 distributions based on the largest absolute distance between empirical distribution functions (e.g., Figures 2A,B); an empirical distribution function is an observed cumulative distribution function. As an extension of the K-S statistic, the K-S measure simultaneously assesses differences in *K* > 2 distributions, using the weighted sum of the K-S statistics for all pairwise comparisons of distributions defined by K groups. The weights are proportional to the number of observations used to calculate each K-S statistic. In this case, to determine the OTUs driving the variation between our *K* = 3 Panamanian frog species, the K-S measure for each OTU was calculated as the weighted sum of the three (3 choose 2) K-S test statistics: (1) comparing *A. callidryas* to *D. ebraccatus*, (2) comparing *A. callidryas* to *C. fitzingeri*, and (3) comparing *D. ebraccatus* to *C. fitzingeri*.

The K-S measure ranges from 0 to 1, where values closer to one imply a greater difference between the K distributions than values closer to zero. K-S measures were calculated for each OTU in our field survey dataset, and the values were plotted in descending order (Figure 2C). We used a natural break in the K-S measures detected after the 46th value as an initial cutoff for OTUs to retain (Loftus et al., 2015). This subset of OTUs was then placed into a multinomial regression model in which species was the response and the selected OTUs were the covariates, and we eliminated an additional 5 of the 46 OTUs that were not significant at a level of *P* = 0.05 for any level of the response. To evaluate how well the final subset of 41 OTUs identified the three species, we used a holdout cross-validation procedure, working with a training sample of 68 randomly chosen frogs, which left a holdout testing sample size of 68 frogs. Of the 68 frogs in the holdout set, we found that the subset of 41 OTUs correctly classified 27/31 *A. callidryas*, 10/10 *C. fitzingeri*, and 20/27 *D. ebraccatus*.

#### Comparison of the diversity of skin microbial communities of amphibians from panamá and the US

We tested for differences in alpha diversity across zones (temperate or tropical) using linear and mixed effect beta regression models as above (linear models for richness and phylogenetic diversity; mixed effect beta regression for Simpson Index). However, when testing for differences between zones, we included a random effect of “site” nested within “species” to account for nestedness of samples of the same species from the same sites.

We assessed if variation in beta diversity was explained by zone using PERMANOVA. We also tested for homogeneity of group dispersions. Community data were converted to distance matrices based on Bray–Curtis dissimilarities. Beta diversity was visualized using NMDS. We then used the K-S measure as described above to determine OTUs that distinguished tropical skin microbiomes from temperate ones. OTUs were sorted in descending order by their K-S measures (Figure 2D), which in this case were equivalent to the K-S statistics because there were only two groups (*K* = 2). We determined a natural cutoff after 31 OTUs. This subset of 31 OTUs perfectly split the tropical and temperate samples when plotted using NMDS (using Bray–Curtis dissimilarities). We explored reducing this number further through multinomial regression, but three of the 31 OTUs only appeared in one of the two zones. This posed a problem for further analysis; a logistic regression model is unidentifiable when including these three OTUs.

#### Impact of Bd on amphibian microbiome structure and function: hypothesis tests

##### Bd infection status

We hypothesized that on individual frogs actively infected with Bd, we would see shifts in the structure and function of the skin microbiome relative to uninfected individuals. To assess if Bd infection status was associated with changes in alpha diversity, we tested for a difference in OTU richness, OTU phylogenetic diversity, and metabolite richness across Bd infected and uninfected individuals using linear mixed models, including a random effect of “site” nested within “species” to account for nestedness of samples of the same species from the same site. To assess whether Bd infection status (infected or not) was associated with changes in OTU community structure or metabolite profiles on individuals, we used PERMANOVA, including a “species × site” term with the strata argument in the adonis model to account for species and site level effects. To assess whether Bd infection decreased variation in community structure or metabolite profiles (i.e., there is strong selection for anti-fungal metabolite production and the taxa that produce them), we tested for homogeneity of group dispersions based on Bd infection status using the function betadisper in the vegan package (Anderson, 2006; Anderson et al., 2006). Betadisper analyses for OTUs (relative abundance) and metabolite profiles (presence/absence) were conducted separately, but, for each of these datasets, using the data for all three species combined. Prior to analysis, OTU and metabolite data were converted to distance matrices based on Bray–Curtis and Jaccard dissimilarities, respectively. To visualize these results, we used NMDS.

##### Bd arrival time across sites

There is evidence that Bd has moved across Panamá from west to east (Lips et al., 2006). Bd was detected near our western most site in January 2007 and is thought to have arrived there in ~2006 (Woodhams et al., 2008). It most likely arrived in our eastern most site in ~2011 (Rebollar et al., 2014), although the precise dates are not known. We hypothesized that this gradient of arrival time should be correlated with aspects of the structure (OTUs) and function (metabolite profiles) of the skin microbiome along the gradient. To test this, we focused on the two treefrog species that were sampled at all four of our sites. For each of these species, we assessed variation in OTU richness, Faith's phylogenetic diversity, and metabolite richness relative to site using linear models. We assessed variation in Simpson Index using beta regression models (package betareg; Cribari-Neto and Zeileis, 2010). If analyses indicated significant differences among sites, we conducted *post-hoc* comparisons, as outlined above for species comparisons, to determine which sites were similar or different from one another. We also assessed changes in OTU community structure and metabolite profiles across this gradient using PERMANOVA. Finally, to test whether the presence of Bd decreases variation in structure and function over time, we assessed homogeneity of group dispersions (function betadisper) in OTU community structure and metabolite profiles along the gradient.

##### Structure-function links and Bd arrival time

We hypothesized that longer-term presence of Bd at a site would drive a stronger correlation between structure and function of the amphibian skin microbiota as selection for strong anti-fungal producing bacterial species occurs over time. Therefore, we expected to see a tighter correlation between OTU community structure and metabolite profiles at our western sites. To assess this, we used Mantel tests to determine if there were correlations between OTU diversity and metabolite diversity for each treefrog species. OTU and metabolite data were converted to Bray–Curtis and Jaccard dissimilarities, respectively. We then compared the strength of the correlation (i.e., the Mantel statistic) across the four sites along our west-east gradient.

## Results

### OTU and metabolite diversity on amphibians from panamá

**Figure 3:**
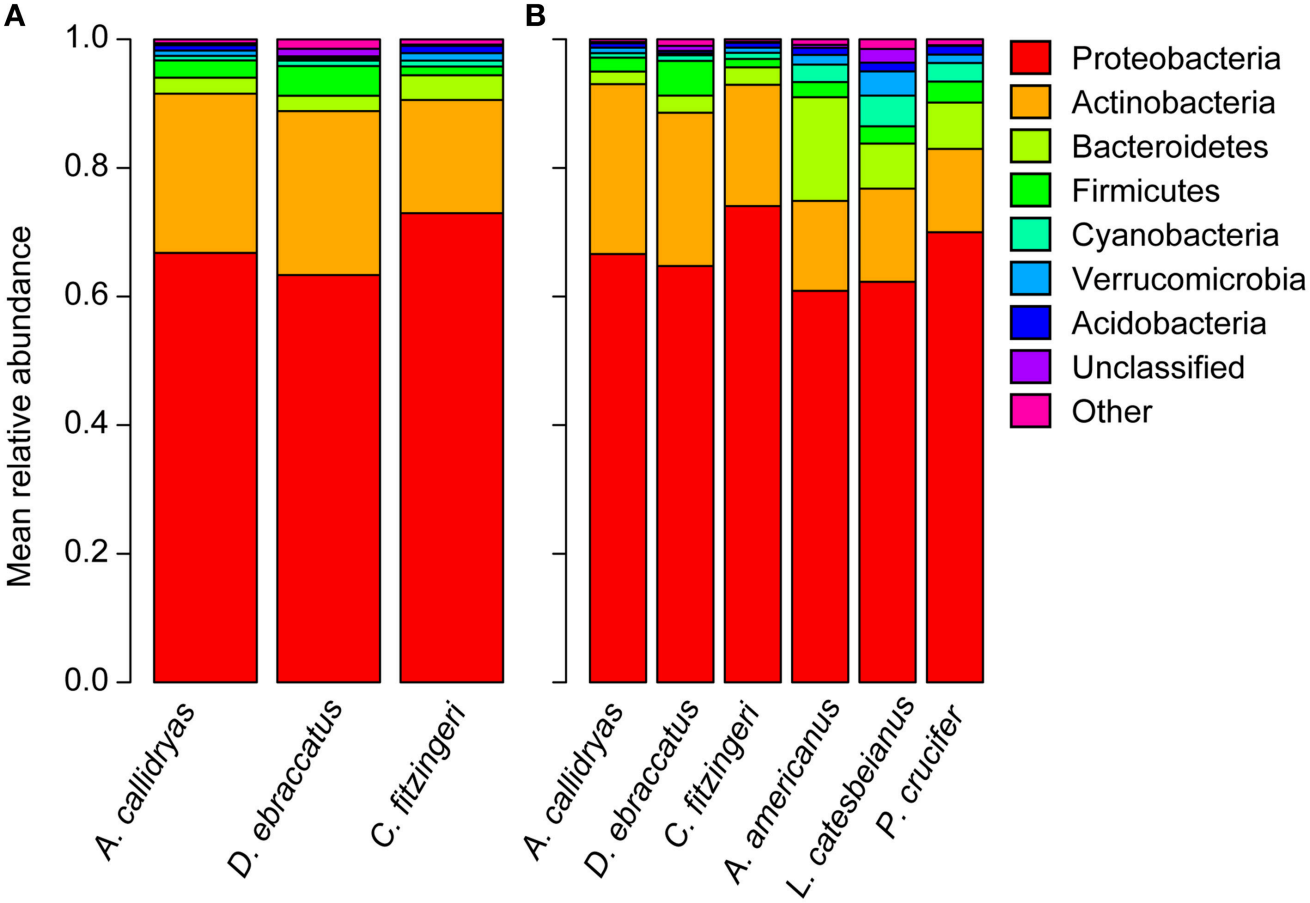
**Mean relative abundance of bacterial phyla present on the skin of (A) three frog species surveyed in Panamá in 2012 (*Agalychnis callidryas, Dendropsophus ebraccatus*, *Craugastor fitzingeri*; this study) and (B) a subset of individuals of each of the three frog species surveyed in Panamá compared to three frog species surveyed in Virginia, USA (*Anaxyrus americanus, Lithobates catesbeianus, Pseudacris crucifer*; Walke et al., 2015; Belden et al. unpublished data)**.

We identified 3138 OTUs from *A. callidryas* (*N* = 62 frogs), 2704 OTUs from *D. ebraccatus* (*N* = 53 frogs), and 2667 OTUs from *C. fitzingeri* (*N* = 21 frogs), based on our 16S rRNA amplicon sequencing. Prominent phyla included Proteobacteria, Actinobacteria, Firmicutes, Bacteroidetes, Cyanobacteria, Verrucomicrobia, and Acidobacteria (Figure 3A).

Alpha diversity differed among the three species, with *C. fitzingeri* harboring the most diverse communities of bacteria in terms of richness (Chisq = 57.3, *P* < 0.001; *post-hoc* comparisons, *A. callidryas*–*D. ebraccatus*, *P* = 0.002; *A. callidryas*–*C. fitzingeri*, *P* < 0.001; *D. ebraccatus*–*C. fitzingeri*, *P* < 0.001; unique OTUs/individual, adjusted mean ± sd: *A. callidryas*, 364 ± 129; *D. ebraccatus*, 290 ± 57; *C. fitzingeri*, 594 ± 202) and phylogenetic diversity (Chisq = 32.4, *P* < 0.001; *post-hoc* comparisons, *A. callidryas*–*D. ebraccatus*, *P* = 0.04; *A. callidryas*–*C. fitzingeri*, *P* < 0.001; *D. ebraccatus*–*C. fitzingeri*, *P* < 0.001; phylogenetic diversity, adjusted mean ± sd: *A. callidryas*, 15.9 ± 5.1; *D. ebraccatus*, 13.7 ± 3.1; *C. fitzingeri*, 22.6 ± 5.3). Although diverse, the bacterial communities of all three species were typically uneven and dominated by a small number of OTUs (Simpson, adjusted mean ± sd: *A. callidryas*, 0.87 ± 0.07; *D. ebraccatus*, 0.88 ± 0.05; *C. fitzingeri*, 0.81 ± 0.08). However, relative to *A. callidryas* and *D. ebraccatus*, the communities of *C. fitzingeri* tended to be more even (as evaluated by Simpson Index; Chisq = 7.9, *P* = 0.02; *post-hoc* comparisons, *A. callidryas*–*D. ebraccatus*, *P* = 0.86; *A. callidryas*–*C. fitzingeri*, *P* = 0.06; *D. ebraccatus*–*C. fitzingeri*, *P* = 0.03).

On average, the top three OTUs in terms of relative abundance accounted for about 50% of the total relative abundance on a given frog (range 9–70%). A comparison of the top three OTUs present on every individual of each species revealed one OTU that dominated the communities of all three species (X394796, Proteobacteria: Pseudomonadaceae; dominant on 88% of individuals; relative abundance/species: mean ± sd: *A. callidryas*, 15 ± 10%, *D. ebraccatus*, 12 ± 9%, *C. fitzingeri*, 32 ± 12%). Two additional OTUs were dominant in the communities of *A. callidryas* and *D. ebraccatus* (X4451011, Proteobacteria: Pseudomonadaceae dominant on 85 and 79% of individuals of *A. callidryas* and *D. ebraccatus*, respectively; relative abundance/species: mean ± sd: *A. callidryas*, 12 ± 7%, *D. ebraccatus*, 13 ± 8%; X235695, Actinobacteria: Cellulomonadaceae dominant on 42 and 57% of individuals of *A. callidryas* and *D. ebraccatus*, respectively; relative abundance/species: mean ± sd: *A. callidryas* 17 ± 19%, *D. ebraccatus* 20 ± 18%). By contrast, *C. fitzingeri* harbored a high abundance of two different OTUs (X4473756, Actinobacteria: Cellulomonadaceae dominant on 76% of individuals; relative abundance, mean ± sd: 7 ± 3% and X1139932, Proteobacteria: Xanthomonadaceae dominant on 67% of individuals; relative abundance, mean ± sd: 6 ± 2%).

**Figure 4:**
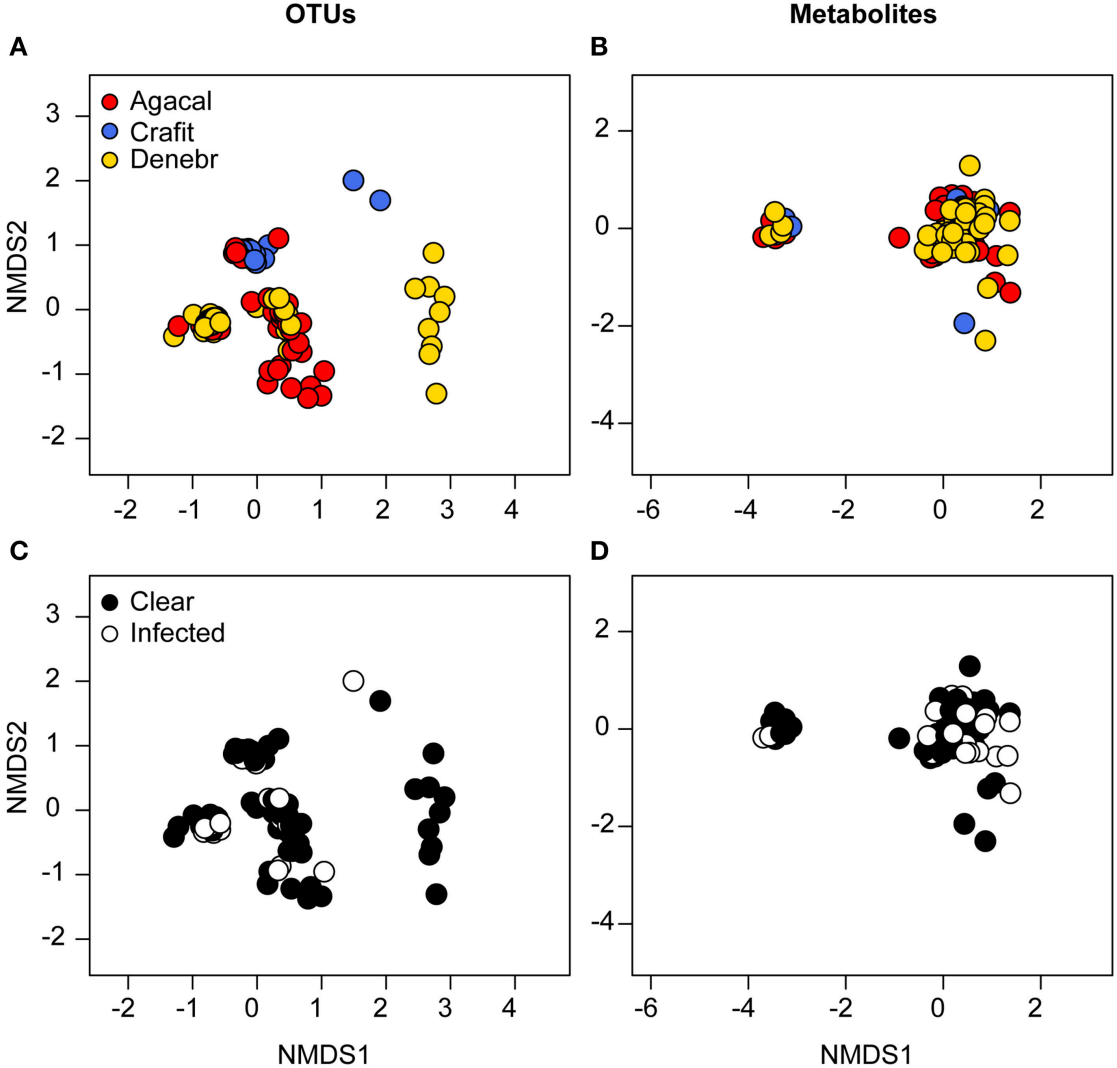
**Beta diversity of bacterial communities (OTUs) (left column) and bacterially-produced metabolite profiles (right column) sampled from the skin of three frog species from Panamá, grouped by frog species (A,B, red, *Agalychnis callidryas*; gold, *Dendropsophus ebraccatus*; blue, *Craugastor fitzingeri*) and the frog's infection status (C,D, white, infected; black, not infected) by the fungal pathogen, *Batrachochytrium dendrobatidis***. NMDS ordinations are based on Bray–Curtis and Jaccard dissimilarities for OTUs and metabolites, respectively. Each point represents a single individual.

**Figure 5:**
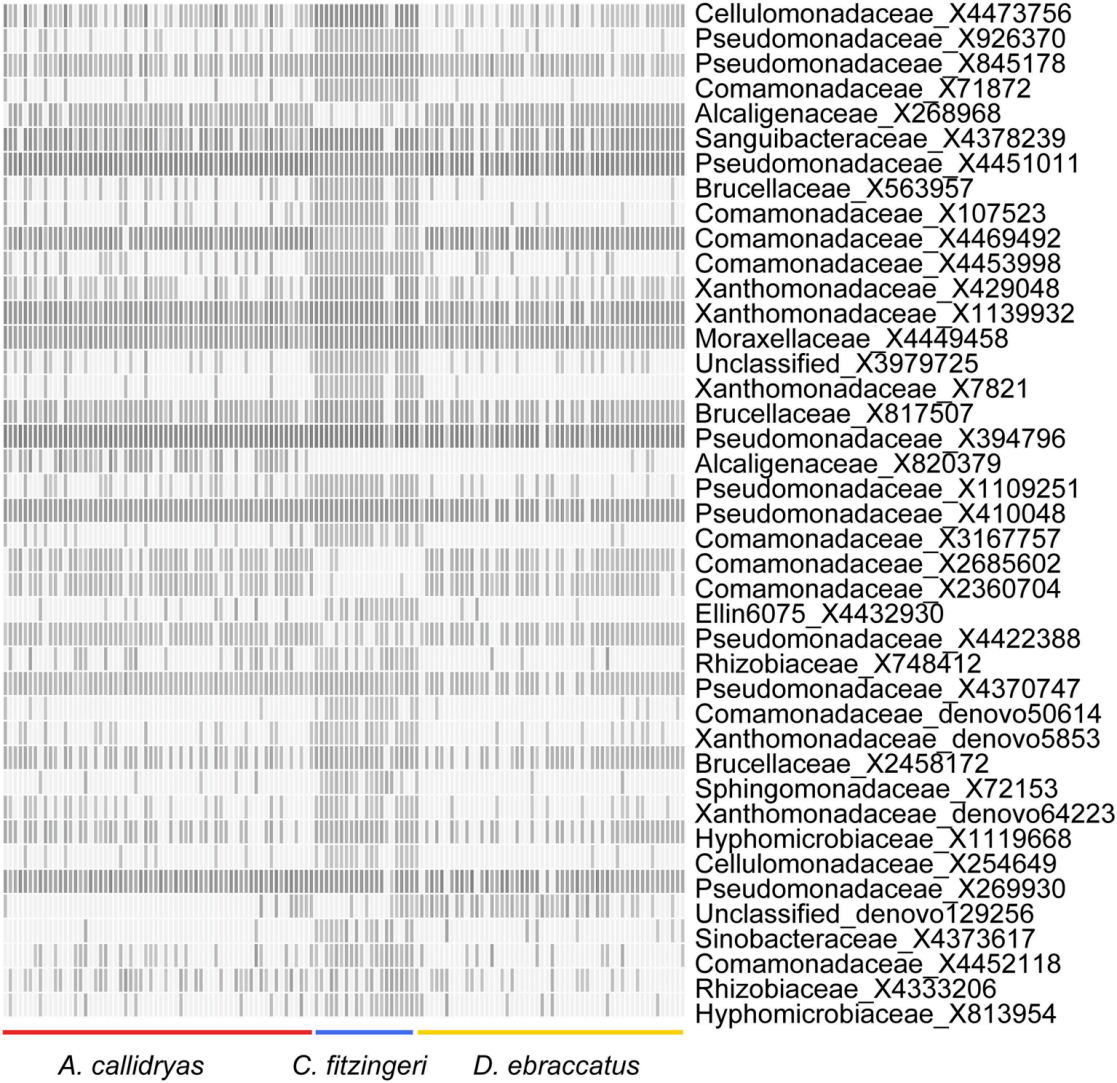
**Relative abundance of 41 OTUs selected based on K-S measures that best defined the differences in skin bacterial community structure of three frog species from Panamá (left, *Agalychnis callidryas*; right, *Dendropsophus ebraccatus*; center, *Craugastor fitzingeri*)**. OTU relative abundances ranged from 0 to 0.32. Lighter shades indicate lower relative abundances (white relative abundance = 0) and darker shades indicate higher relative abundances (darkest relative abundance = 0.32). OTUs are ordered top to bottom based on K-S measures (see Table 2 for exact values and additional taxonomic information for each OTU).

Based on NMDS ordination, there were differences in OTU community structure among the three frog species when accounting for site (Figure 4A, NMDS stress: 0.11, Adonis pseudo = 12.92, *R*^2^ = 0.16, *P* = 0.001; *post-hoc* comparisons, *A. callidryas*–*D. ebraccatus* Adonis pseudo = 2.43, *R*^2^ = 0.02, *P* = 0.008; *A. callidryas*–*C. fitzingeri* Adonis pseudoF = 20.4, *R*^2^ = 0.20, *P* = 0.001; *D. ebraccatus*–*C. fitzingeri* Adonis pseudoF = 23.2, *R*^2^ = 0.24, *P* = 0.001). K-S measures of the final set of 41 species-defining OTUs ranged from 0.40 to 0.60 (Table 2). Samples of *C. fitzingeri* were clearly distinguishable based on these OTUs; many K-S-defined OTUs that commonly occurred in high abundance in samples of *C. fitzingeri* were rare on the two treefrogs, and vice versa (i.e., OTUs that were rare on *C. fitzingeri* were abundant on the treefrogs; Figure 5). Differences between the treefrogs were more subtle. Exceptions include an OTU in the family Alcaligenaceae (X820379), found almost exclusively on *A. callidryas*, and an unclassified OTU in the candidate phylum GN02 (denovo129256) that was abundant in samples of *D. ebraccatus* (Figure 5).

**Table 2:** **K-S Measures and taxonomic information for 41 OTUs that best defined the differences in skin bacterial community structure of three frog species from Panamá: *Agalychnis callidryas, Dendropsophus ebraccatus*, and *Craugastor fitzingeri***.

**OTU**	**K-S measure**	**Phylum**	**Class**	**Order**	**Family**	**Genus**
X4473756	0.60	Actinobacteria	Actinobacteria	Actinomycetales	Cellulomonadaceae	Unclassified
X926370	0.59	Proteobacteria	Gammaproteobacteria	Pseudomonadales	Pseudomonadaceae	*Pseudomonas*
X845178	0.59	Proteobacteria	Gammaproteobacteria	Pseudomonadales	Pseudomonadaceae	Unclassified
X71872	0.59	Proteobacteria	Betaproteobacteria	Burkholderiales	Comamonadaceae	*Comamonas*
X268968	0.59	Proteobacteria	Betaproteobacteria	Burkholderiales	Alcaligenaceae	*Achromobacter*
X4378239	0.58	Actinobacteria	Actinobacteria	Actinomycetales	Sanguibacteraceae	*Sanguibacter*
X4451011	0.58	Proteobacteria	Gammaproteobacteria	Pseudomonadales	Pseudomonadaceae	Unclassified
X563957	0.58	Proteobacteria	Alphaproteobacteria	Rhizobiales	Brucellaceae	Unclassified
X107523	0.58	Proteobacteria	Betaproteobacteria	Burkholderiales	Comamonadaceae	Unclassified
X4469492	0.55	Proteobacteria	Betaproteobacteria	Burkholderiales	Comamonadaceae	Unclassified
X4453998	0.55	Proteobacteria	Betaproteobacteria	Burkholderiales	Comamonadaceae	Unclassified
X429048	0.55	Proteobacteria	Gammaproteobacteria	Xanthomonadales	Xanthomonadaceae	Unclassified
X1139932	0.55	Proteobacteria	Gammaproteobacteria	Xanthomonadales	Xanthomonadaceae	Unclassified
X4449458	0.55	Proteobacteria	Gammaproteobacteria	Pseudomonadales	Moraxellaceae	*Acinetobacter*
X3979725	0.54	Actinobacteria	Actinobacteria	Actinomycetales	Unclassified	Unclassified
X7821	0.52	Proteobacteria	Gammaproteobacteria	Xanthomonadales	Xanthomonadaceae	*Stenotrophomonas acidaminiphila*
X817507	0.52	Proteobacteria	Alphaproteobacteria	Rhizobiales	Brucellaceae	Unclassified
X394796	0.52	Proteobacteria	Gammaproteobacteria	Pseudomonadales	Pseudomonadaceae	*Pseudomonas viridiflava*
X820379	0.51	Proteobacteria	Betaproteobacteria	Burkholderiales	Alcaligenaceae	Unclassified
X1109251	0.50	Proteobacteria	Gammaproteobacteria	Pseudomonadales	Pseudomonadaceae	*Pseudomonas*
X410048	0.49	Proteobacteria	Gammaproteobacteria	Pseudomonadales	Pseudomonadaceae	*Pseudomonas*
X3167757	0.48	Proteobacteria	Betaproteobacteria	Burkholderiales	Comamonadaceae	Unclassified
X2685602	0.47	Proteobacteria	Betaproteobacteria	Burkholderiales	Comamonadaceae	Unclassified
X2360704	0.46	Proteobacteria	Betaproteobacteria	Burkholderiales	Comamonadaceae	Unclassified
X4432930	0.45	Acidobacteria	[Chloracidobacteria]	RB41	Ellin6075	Unclassified
X4422388	0.45	Proteobacteria	Gammaproteobacteria	Pseudomonadales	Pseudomonadaceae	Unclassified
X748412	0.44	Proteobacteria	Alphaproteobacteria	Rhizobiales	Rhizobiaceae	*Agrobacterium*
X4370747	0.44	Proteobacteria	Gammaproteobacteria	Pseudomonadales	Pseudomonadaceae	*Pseudomonas viridiflava*
denovo50614	0.43	Proteobacteria	Betaproteobacteria	Burkholderiales	Comamonadaceae	Unclassified
denovo5853	0.43	Proteobacteria	Gammaproteobacteria	Xanthomonadales	Xanthomonadaceae	Unclassified
X2458172	0.43	Proteobacteria	Alphaproteobacteria	Rhizobiales	Brucellaceae	*Ochrobactrum*
X72153	0.42	Proteobacteria	Alphaproteobacteria	Sphingomonadales	Sphingomonadaceae	Unclassified
denovo64223	0.42	Proteobacteria	Gammaproteobacteria	Xanthomonadales	Xanthomonadaceae	Unclassified
X1119668	0.42	Proteobacteria	Alphaproteobacteria	Rhizobiales	Hyphomicrobiaceae	*Devosia*
X254649	0.42	Actinobacteria	Actinobacteria	Actinomycetales	Cellulomonadaceae	Unclassified
X269930	0.42	Proteobacteria	Gammaproteobacteria	Pseudomonadales	Pseudomonadaceae	*Pseudomonas veronii*
denovo129256	0.42	GN02	BD1-5	Unclassified	Unclassified	Unclassified
X4373617	0.41	Proteobacteria	Gammaproteobacteria	Xanthomonadales	Sinobacteraceae	Unclassified
X4452118	0.41	Proteobacteria	Betaproteobacteria	Burkholderiales	Comamonadaceae	Unclassified
X4333206	0.40	Proteobacteria	Alphaproteobacteria	Rhizobiales	Rhizobiaceae	*Agrobacterium*
X813954	0.40	Proteobacteria	Alphaproteobacteria	Rhizobiales	Hyphomicrobiaceae	*Devosia*

The K-S Measure ranges from 0 to 1, where values closer to one imply a greater difference between the K distributions than values closer to zero. OTUs are listed in order of descending K-S Measure. Most OTUs were unclassified at the species level; if species information was available, it is listed along with the genus in the Genus column.

We identified a total of 163 unique metabolites from the skin of our three frog species from Panamá (total number of metabolites/species: *A. callidryas*, 135; *D. ebraccatus*, 120; *C. fitzingeri*, 105) based on the HPLC-MS analysis. Metabolite richness differed across the three species (Chisq = 6.3, *P* = 0.04). Richness values were on average highest for *A. callidryas* and lowest for *D. ebraccatus* (*post-hoc* comparisons: *A. callidryas*–*C. fitzingeri*, *P* = 0.6; *A. callidryas*–*D. ebraccatus*, *P* = 0.03; *D. ebraccatus*–*C. fitzingeri*, *P* = 0.6; richness, adjusted mean ± sd: *A. callidryas*, 32 ± 7; *D. ebraccatus*, 28 ± 8; *C. fitzingeri*, 30 ± 8); however, the number of metabolites associated with any given individual was variable for all three species (range: 13–47 metabolites for *A. callidryas*, 8–50 for *D. ebraccatus*, and 16–52 for *C. fitzingeri*). Metabolite profiles also differed among the three species when accounting for site (Figure 4B, NMDS stress: 0.12, Adonis pseudoF = 1.9, *R*^2^ = 0.03, *P* = 0.02; *post-hoc* comparisons, *A. callidryas*–*D. ebraccatus* Adonis pseudoF = 2.2, *R*^2^ = 0.02, *P* = 0.01; *A. callidryas*–*C. fitzingeri* Adonis pseudoF = 2.3, *R*^2^ = 0.03, *P* = 0.05; *D. ebraccatus*–*C. fitzingeri* Adonis pseudoF = 1.16, *R*^2^ = 0.02, *P* = 0.35), although the separation was not as clear on the NMDS as it was for the OTUs (PERMANOVA: *R*^2^ = 0.03 for metabolites vs. *R*^2^ = 0.16 for OTUs).

### Comparison of the diversity of skin microbial communities of amphibians from panamá and the US

Despite wide geographic separation and likely variation in the history of Bd presence, at the phylum level, similar taxa dominated the skin bacterial communities of amphibians from Panamá and the US. The Proteobacteria accounted for over 60% of the relative abundance of the bacterial assemblages for all species (Figure 3B; relative abundance, mean ± sd: *A. callidryas* 67 ± 15%, *D. ebraccatus* 65 ± 15%, *C. fitzingeri* 74 ± 5%, *A. americanus* 61 ± 9%, *L. catesbeianus* 62 ± 13%, *P. crucifer* 70 ± 15%). The Actinobacteria accounted for the second largest proportion of the relative abundance of all three species from Panamá, whereas the Actinobacteria and Bacteroidetes were more evenly represented in terms of relative abundance for the three species from the US (Figure 3B).

In terms of richness and phylogenetic diversity, the bacterial communities on the skin of the temperate and tropical amphibians we examined were similar (richness, Chisq = 2.9, *P* = 0.09; unique OTUs/individual, adjusted mean ± sd: Temperate species 609 ± 279; Tropical species, 401 ± 165; phylogenetic diversity, Chisq = 0.216, *P* = 0.64, adjusted mean ± sd: Temperate species 19.7 ± 7.8; Tropical species, 16.3 ± 4.4). Overall, the communities of tropical species were slightly more even than those of temperate species (Simpson Index, Chisq = 4.3, *P* = 0.04, adjusted mean ± sd: Temperate species 0.88 ± 0.09; Tropical species, 0.86 ± 0.05).

**Figure 6:**
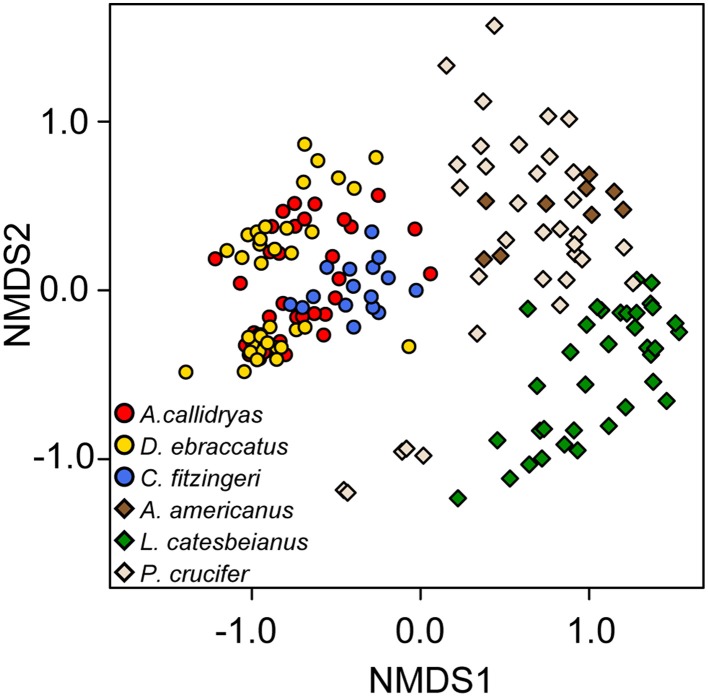
**Beta diversity of skin bacterial communities of tropical (circles) and temperate (diamonds) amphibians**. Three species from each zone were included in the analysis [red, *Agalychnis callidryas*; gold, *Dendropsophus ebraccatus*; blue, *Craugastor fitzingeri* from Panamá (tropical zone); brown, *Anaxyrus americanus*; green, *Lithobates catesbeianus*; cream, *Pseudacris crucifer* from Virginia, USA (temperate zone)]. NMDS ordination based on Bray–Curtis dissimilarities. Each point represents a single individual.

**Figure 7:**
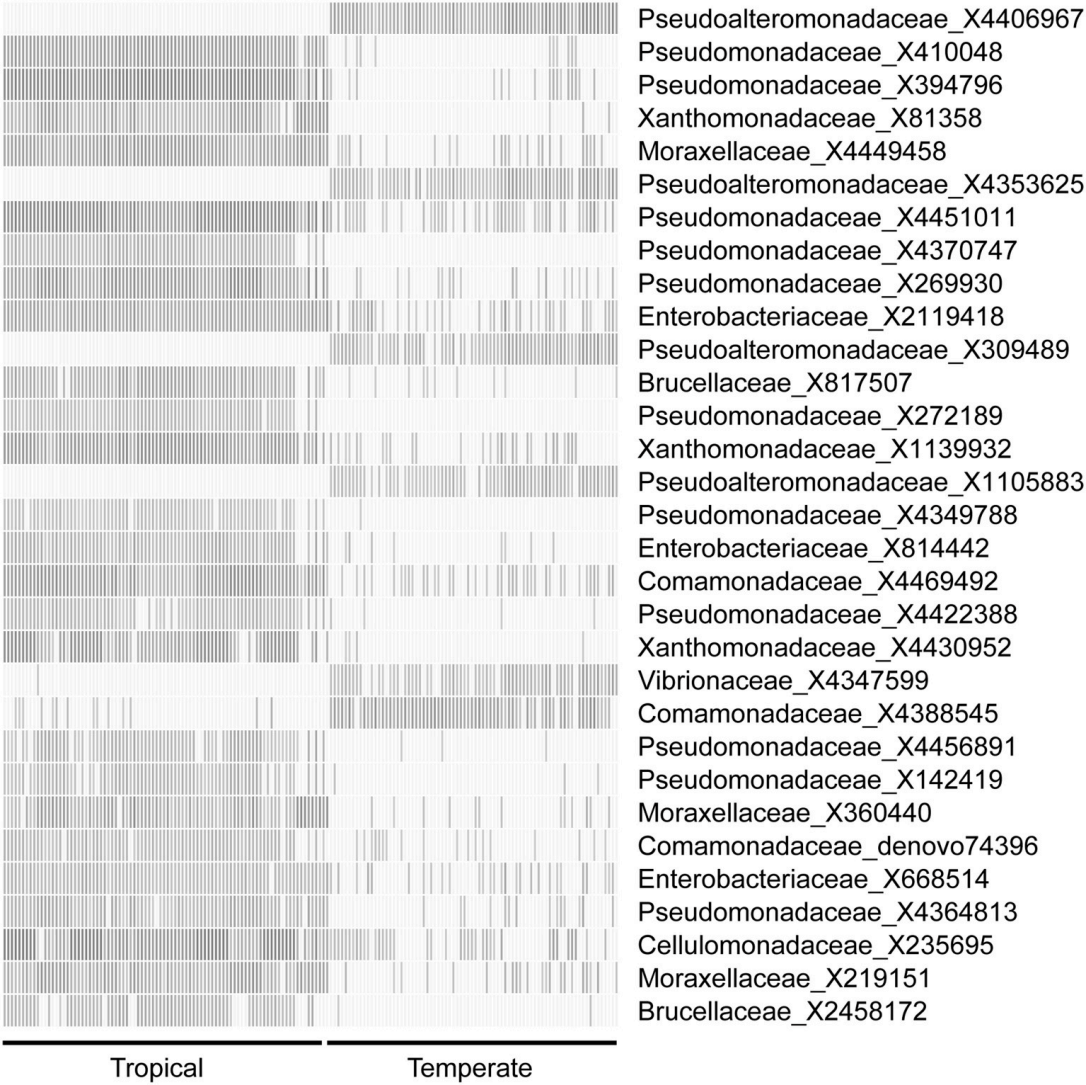
**Relative abundance of 31 OTUs selected based on K-S measures that best defined the differences in skin bacterial community structure of tropical (left) and temperate (right) amphibians**. Three species from each zone were included in the analysis: *Agalychnis callidryas, Dendropsophus ebraccatus, Craugastor fitzingeri* from Panamá (tropical zone) and *Anaxyrus americanus, Lithobates catesbeianus, Pseudacris crucifer* from Virginia, USA (temperate zone). OTU relative abundances ranged from 0 to 0.37. Lighter shades indicate lower relative abundances (white, relative abundance = 0) and darker shades indicate higher relative abundances (darkest relative abundance = 0.37). OTUs are ordered top to bottom based on K-S measures (see Table 3 for exact values and additional taxonomic information for each OTU).

While 68% (3228/4698) of the OTUs in this dataset were found on frogs in both the temperate and tropical zones, the skin bacterial communities of temperate and tropical amphibians were distinct (Figure 6, NMDS stress: 0.15, Adonis pseudoF = 49.73, *R*^2^ = 0.23, *P* = 0.001). Tropical and temperate frog species clustered separately on the NMDS plot, but they also varied in dispersion, with temperate frogs seeming to have much more variance in the skin bacterial community as compared to the tropical frogs (test of beta-dispersion, *F* = 84.56, *P* = 0.001). Given these differences, we used the K-S measure to identify some of key OTUs that contributed the most to this difference among the temperate and tropical frogs we sampled. K-S measures for the final set of 31 OTUs defining tropical and temperate species ranged from 0.38 to 0.50 (Table 3). Most of the K-S defined OTUs were present in both zones, but exhibited higher relative abundance distributions for the tropical species (Figure 7). However, several OTUs exhibited complete separation by zone (Figure 7). Specifically, two OTUs in the family Pseudomonadaceae, X4370747 and X272189, were unique to tropical species, whereas four OTUs in the family Pseudoalteromonadaceae—X4406967, X1105883, X309489, and X4353625—were only found on temperate species (Figure 7).

**Table 3:** **K-S Measures and taxonomic information for 31 OTUs that best defined the differences in skin bacterial community structure of tropical and temperate amphibians**.

**OUT**	**K-S measure**	**Phylum**	**Class**	**Order**	**Family**	**Genus**
X4406967	0.50	Proteobacteria	Gammaproteobacteria	Vibrionales	Pseudoalteromonadaceae	*Pseudoalteromonas*
X410048	0.48	Proteobacteria	Gammaproteobacteria	Pseudomonadales	Pseudomonadaceae	Unclassified
X394796	0.48	Proteobacteria	Gammaproteobacteria	Pseudomonadales	Pseudomonadaceae	*Pseudomonas viridiflava*
X81358	0.48	Proteobacteria	Gammaproteobacteria	Xanthomonadales	Xanthomonadaceae	*Rhodanobacter*
X4449458	0.48	Proteobacteria	Gammaproteobacteria	Pseudomonadales	Moraxellaceae	*Acinetobacter*
X4353625	0.47	Proteobacteria	Gammaproteobacteria	Vibrionales	Pseudoalteromonadaceae	*Pseudoalteromonas*
X4451011	0.47	Proteobacteria	Gammaproteobacteria	Pseudomonadales	Pseudomonadaceae	Unclassified
X4370747	0.47	Proteobacteria	Gammaproteobacteria	Pseudomonadales	Pseudomonadaceae	*Pseudomonas viridiflava*
X269930	0.47	Proteobacteria	Gammaproteobacteria	Pseudomonadales	Pseudomonadaceae	*Pseudomonas veronii*
X2119418	0.46	Proteobacteria	Gammaproteobacteria	Enterobacteriales	Enterobacteriaceae	Unclassified
X309489	0.46	Proteobacteria	Gammaproteobacteria	Vibrionales	Pseudoalteromonadaceae	*Pseudoalteromonas*
X817507	0.45	Proteobacteria	Alphaproteobacteria	Rhizobiales	Brucellaceae	Unclassified
X272189	0.45	Proteobacteria	Gammaproteobacteria	Pseudomonadales	Pseudomonadaceae	*Pseudomonas viridiflava*
X1139932	0.45	Proteobacteria	Gammaproteobacteria	Xanthomonadales	Xanthomonadaceae	Unclassified
X1105883	0.44	Proteobacteria	Gammaproteobacteria	Vibrionales	Pseudoalteromonadaceae	*Pseudoalteromonas*
X4349788	0.44	Proteobacteria	Gammaproteobacteria	Pseudomonadales	Pseudomonadaceae	*Pseudomonas viridiflava*
X814442	0.44	Proteobacteria	Gammaproteobacteria	Enterobacteriales	Enterobacteriaceae	*Citrobacter*
X4469492	0.43	Proteobacteria	Betaproteobacteria	Burkholderiales	Comamonadaceae	Unclassified
X4422388	0.42	Proteobacteria	Gammaproteobacteria	Pseudomonadales	Pseudomonadaceae	Unclassified
X4430952	0.42	Proteobacteria	Gammaproteobacteria	Xanthomonadales	Xanthomonadaceae	*Stenotrophomonas*
X4347599	0.42	Proteobacteria	Gammaproteobacteria	Vibrionales	Vibrionaceae	*Vibrio*
X4388545	0.41	Proteobacteria	Betaproteobacteria	Burkholderiales	Comamonadaceae	*Rhodoferax*
X4456891	0.41	Proteobacteria	Gammaproteobacteria	Pseudomonadales	Pseudomonadaceae	*Pseudomonas*
X142419	0.41	Proteobacteria	Gammaproteobacteria	Pseudomonadales	Pseudomonadaceae	*Pseudomonas*
X360440	0.40	Proteobacteria	Gammaproteobacteria	Pseudomonadales	Moraxellaceae	*Acinetobacter rhizosphaerae*
denovo74396	0.40	Proteobacteria	Betaproteobacteria	Burkholderiales	Comamonadaceae	Unclassified
X668514	0.39	Proteobacteria	Gammaproteobacteria	Enterobacteriales	Enterobacteriaceae	Unclassified
X4364813	0.39	Proteobacteria	Gammaproteobacteria	Pseudomonadales	Pseudomonadaceae	Unclassified
X235695	0.39	Actinobacteria	Actinobacteria	Actinomycetales	Cellulomonadaceae	*Cellulomonas*
X219151	0.38	Proteobacteria	Gammaproteobacteria	Pseudomonadales	Moraxellaceae	*Acinetobacter*
X2458172	0.38	Proteobacteria	Alphaproteobacteria	Rhizobiales	Brucellaceae	*Ochrobactrum*

The K-S Measure ranges from 0 to 1, where values closer to one imply a greater difference between the K distributions than values closer to zero. Three species from each zone were included in the analysis: Agalychnis callidryas, Dendropsophus ebraccatus, Craugastor fitzingeri from Panamá (tropical zone) and Anaxyrus americanus, Lithobates catesbeianus, Pseudacris crucifer from Virginia, USA (temperate zone). OTUs are listed in order of descending K-S Measure. Most OTUs were unclassified at the species level; if species information was available, it is listed along with the genus in the Genus column.

### Impact of Bd on amphibian microbiome structure and function: hypothesis tests

#### Bd infection status

A total of 35 frogs were infected with Bd in our survey, with 3–4 individuals infected/species/site on average (details in Table 2 in Rebollar et al., 2014). Treefrog *Bd* infection intensities (zoospore equivalents) across the four sites varied from 0.09 to 0.51 for the 16 infected *A. callidryas* and 0.15–0.24 for the 12 infected *D. ebraccatus* (reported in Table 2 in Rebollar et al., 2014). In addition, Bd infection intensities for the seven infected *C. fitzingeri* across two sites varied from 0.05 to 36.56 (reported in Table 2 in Rebollar et al., 2014). Bd infection status of individuals was not associated with changes in alpha diversity of either OTUs or metabolite profiles (OTU richness: Chisq = 0.02, *P* = 0.9; phylogenetic diversity: Chisq = 0.01, *P* = 0.9; metabolite richness: Chisq = 1.2, *P* = 0.3). Bd-infected individuals also did not cluster separately from uninfected individuals based on NMDS analyses of the structure (OTUs) and function (metabolite profiles) of the skin microbial communities after accounting for “species × site” level effects (OTUs, Figure 4C, NMDS stress: 0.11, Adonis pseudoF = 2.6, *R*^2^ = 0.05, *P* = 0.23; metabolites, Figure 4D, NMDS stress: 0.12, Adonis pseudoF = 0.81, *R*^2^ = 0.01, *P* = 0.76). Some caution should be used when inferring from these results relating to Bd infection status due to low statistical power when accounting for species and site level effects in the models. For example, approximate power calculations for the Bd effect size in richness when accounting for site and species are on the order of 10–20%. Thus, if we were to sample frogs with the same rate of infection (~1 Bd-infected frog among 5 frogs), we would need to sample >350 frogs to improve our power to 80%.

#### Bd arrival time across sites

**Figure 8:**
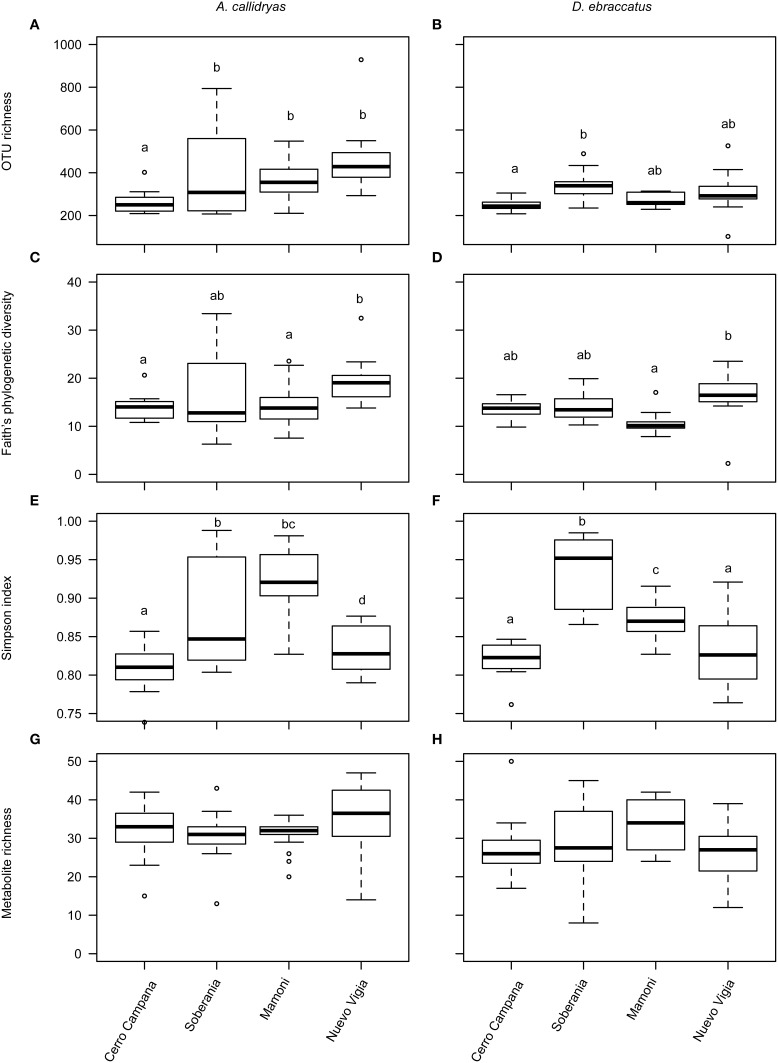
**Alpha diversity of bacterial OTUs (A,B, richness; C,D, phylogenetic diversity; E,F, evenness) and bacterially-produced metabolites (G,H, richness) sampled from the skin of *Agalychnis callidryas* (left column) and *Dendropsophus ebraccatus* (right column)**. Both species were sampled at each of four locations during a field survey conducted in Panamá in 2012. Sites are ordered left to right along the x-axis based on the relative length of time these species have persisted with the fungal pathogen, *Batrachochytrium dendrobatidis*, at each site (i.e., longest at Cerro Campana and shortest at Nuevo Vigia; Woodhams et al., 2008; Rebollar et al., 2014). The letters above the bars indicate statistically significant differences among sites.

Alpha-diversity of skin bacterial communities, in terms of OTU richness, phylogenetic diversity, and evenness varied significantly across the four sites for both species of treefrogs (Figures 8A–F, *A. callidryas*, OTU richness: *F* = 6.75, *P* < 0.001, phylogenetic diversity: *F* = 3.55, *P* = 0.02, Simpson index: Chisq = 53.3, *P* < 0.001; *D. ebraccatus*, OTU richness: *F* = 4.46, *P* = 0.007, phylogenetic diversity: *F* = 6.01, *P* = 0.001, Simpson index: Chisq = 69.9, *P* < 0.001). Metabolite richness did not vary across sites (Figures 8G,H, *A. callidryas*, *F* = 1.11, *P* = 0.35; *D. ebraccatus*, *F* = 1.33, *P* = 0.27). However, while there was variation in OTU alpha-diversity across sites, the variation was not consistent with changes that would have been expected with the movement of Bd from western to eastern sites. For *A. callidryas*, OTU richness and phylogenetic diversity was highest at the eastern most site (most recent Bd arrival, Nuevo Vigia; *post-hoc* tests summarized in Table 4). However, this same pattern was not seen in *D. ebraccatus*, where OTU richness only differed between the two western most sites, and phylogenetic diversity only differed between the two eastern most sites (*post-hoc* tests summarized in Table 4). For both treefrog species, the two central sites tended to have communities dominated by few OTUs, with more even communities at the western and eastern sites (*post-hoc* tests summarized in Table 4).

**Table 4:** **Results of *post-hoc* comparisons indicating if OTU alpha diversity measures (richness, phylogenetic diversity, Simpson Index) were similar or different among four sites sampled during a field survey assessing the diversity of bacterial communities on amphibian skin**.

	**Estimate**	**Std. Error**	**Test statistic**	***P*-value**
***A. callidryas* RICHNESS**				
Soberania—CerroCampana	0.33	0.119	2.778	**0.036**
Mamoni—CerroCampana	0.336	0.111	3.026	**0.019**
NuevoVigia—CerroCampana	0.551	0.126	4.372	**< 0.001**
Mamoni—Soberania	0.006	0.111	0.056	0.999
NuevoVigia—Soberania	0.221	0.126	1.753	0.3055
NuevoVigia—Mamoni	0.215	0.118	1.807	0.28
***D. ebraccatus* RICHNESS**				
Soberania—CerroCampana	0.293	0.082	3.554	**0.005**
Mamoni—CerroCampana	0.079	0.944	0.845	0.832
NuevoVigia—CerroCampana	0.156	0.081	1.929	0.229
Mamoni—Soberania	−0.214	0.948	−2.258	0.122
NuevoVigia—Soberania	−0.137	0.082	−1.659	0.355
NuevoVigia—Mamoni	0.077	0.093	0.826	0.841
***A. callidryas* PHYLOGENETIC DIVERSITY**				
Soberania—CerroCampana	2.569	1.853	1.386	0.512
Mamoni—CerroCampana	0.267	1.734	0.169	0.998
NuevoVigia—CerroCampana	5.583	1.966	2.841	**0.031**
Mamoni—Soberania	−2.276	1.734	−1.313	0.558
NuevoVigia—Soberania	3.015	1.966	1.534	0.424
NuevoVigia—Mamoni	5.291	1.853	2.855	0.294
***D. ebraccatus* PHYLOGENETIC DIVERSITY**				
Soberania—CerroCampana	0.464	1.217	0.382	0.981
Mamoni—CerroCampana	−2.763	1.381	−2.001	0.201
NuevoVigia—CerroCampana	3.002	1.196	2.511	0.07
Mamoni—Soberania	−3.227	1.399	−2.307	0.11
NuevoVigia—Soberania	2.538	1.217	2.085	0.717
NuevoVigia—Mamoni	5.765	1.381	4.175	**< 0.001**
***A. callidryas* SIMPSON INDEX**				
Soberania—CerroCampana			13.4	**< 0.001**
Mamoni—CerroCampana			38.7	**< 0.001**
NuevoVigia—CerroCampana			4.7	**0.03**
Mamoni—Soberania			1.0	0.3
NuevoVigia—Soberania			7.0	**0.008**
NuevoVigia—Mamoni			24.9	**< 0.001**
***D. ebraccatus* SIMPSON INDEX**				
Soberania—CerroCampana			33.2	**< 0.001**
Mamoni—CerroCampana			18.2	**< 0.001**
NuevoVigia—CerroCampana			1.6	0.2
Mamoni—Soberania			11.9	**< 0.001**
NuevoVigia—Soberania			23.3	**< 0.001**
NuevoVigia—Mamoni			3.7	**0.05**

Results are presented for the two species, Agalychnis callidryas and Dendropsophus ebraccatus, that were sampled at all four sites. For richness and phylogenetic diversity the reported test statistic is t, while for Simpson Index the reported test statistic is Chi-Square. Bolded P-values are statistically significant.

**Figure 9:**
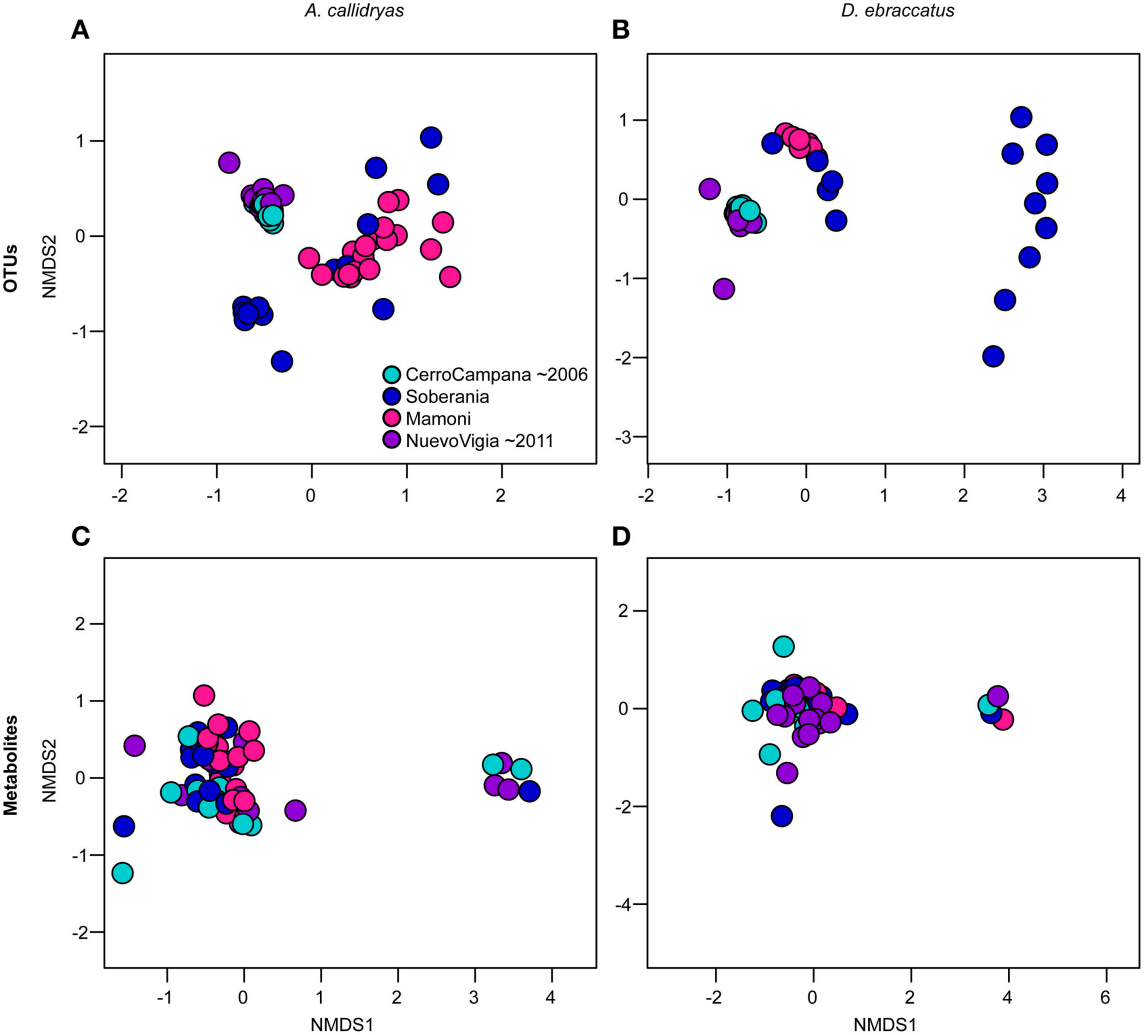
**Beta diversity of bacterial communities (OTUs) (A,B) and bacterially-produced metabolite profiles (C,D) sampled from the skin *Agalychnis callidryas* (left column), *Dendropsophus ebraccatus* (right column), grouped by sampling location**. Both species were sampled at each of four locations during a field survey conducted in Panamá in 2012. The color of the circles indicates the four sites and relative length of time these species have persisted with the fungal pathogen, *Batrachochytrium dendrobatidis*, at each site (i.e., aquamarine = longest at Cerro Campana and purple = shortest at Nuevo Vigia; Woodhams et al., 2008; Rebollar et al., 2014). NMDS ordinations are based on Bray–Curtis and Jaccard dissimilarities for OTUs and metabolites, respectively. Each point represents a single individual.

Beta-diversity of OTUs varied across the four sites for both treefrog species, while metabolite profiles only differed across sites for *A. callidryas* (OTUs: A. callidryas, Figure 9A, NMDS stress: 0.12, Adonis pseudoF = 19.25, *R*^2^ = 0.50, *P* = 0.001; *D. ebraccatus*, Figure 9B, NMDS stress: 0.06, Adonis pseudoF = 20.24, *R*^2^ = 0.55, *P* = 0.001; Metabolites: *A. callidryas*, Figure 9C, NMDS stress: 0.12, Adonis pseudoF = 2.17, *R*^2^ = 0.10, *P* = 0.001; *D. ebraccatus*, Figure 9D, NMDS stress: 0.11, Adonis pseudoF = 1.40, *R*^2^ = 0.08, *P* = 0.07). However, as for the alpha-diversity metrics, there was no clear indication that this variation across sites was strongly associated with the west-east gradient of Bd arrival times. For both treefrog species, the individuals from the two sites farthest west and east on our gradient (Cerro Campana and Nuevo Vigia) clustered together on the NMDS plots of OTU communities and were separate from the two central sites (Figures 9A,B).

**Figure 10:**
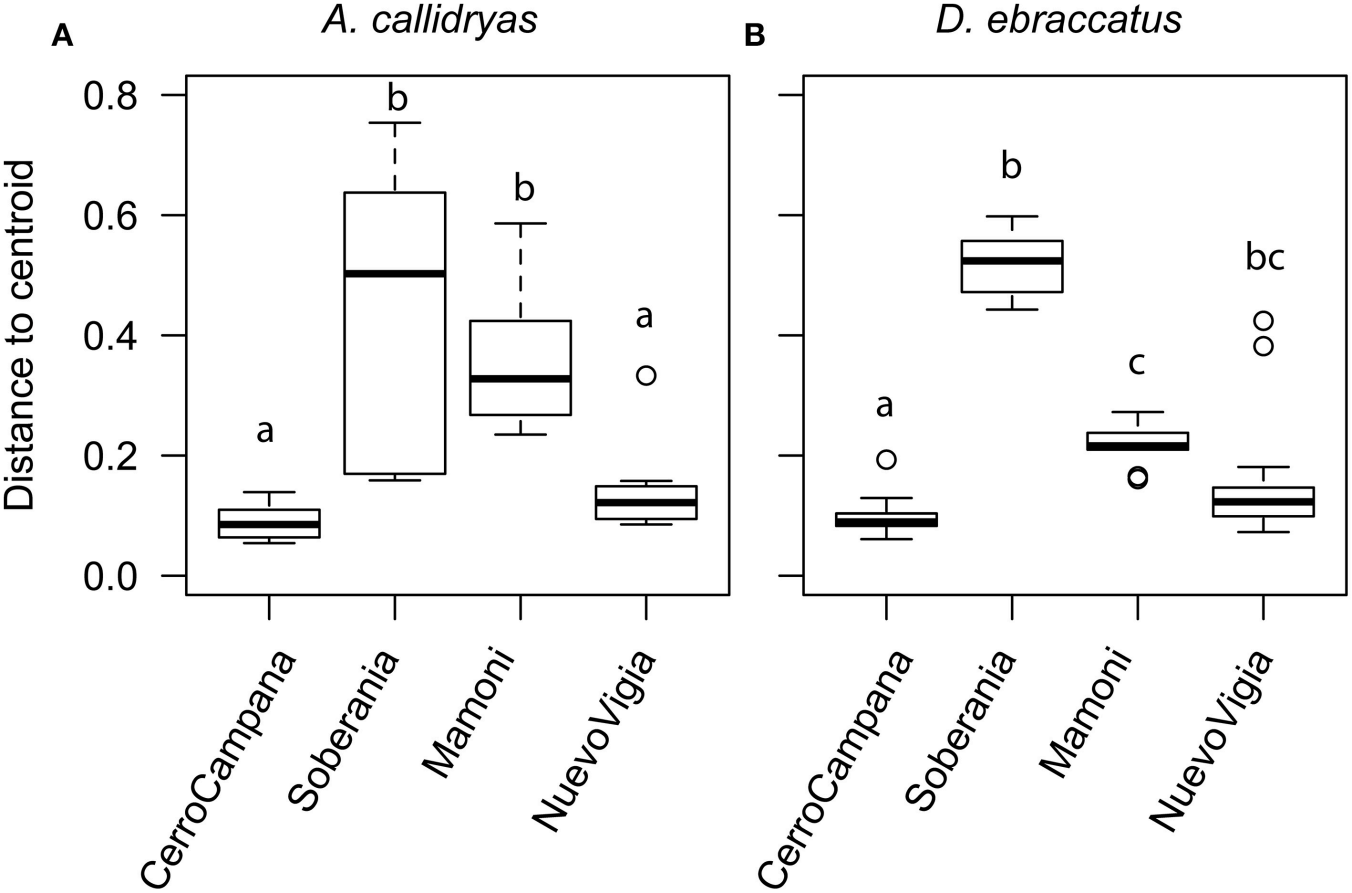
**Dispersion of the bacterial communities (OTUs) sampled from the skin of (A) *Agalychnis callidryas* and (B) *Dendropsophus ebraccatus***. Both species were sampled at each of four locations during a field survey conducted in Panamá in 2012. Sites are ordered left to right along the x-axis based on the relative length of time these species have persisted with the fungal pathogen, *Batrachochytrium dendrobatidis*, at each site (i.e., longest at Cerro Campana and shortest at Nuevo Vigia; Woodhams et al., 2008; Rebollar et al., 2014). Analyses are based on Bray–Curtis and Jaccard dissimilarities for OTUs and metabolites, respectively. The letters above the bars indicate statistically significant differences among sites.

We also examined whether variance (dispersion) in the OTU communities or metabolite profiles varied across sites. We found that for both treefrog species, dispersion did differ among sites for the OTUs (Figures 10A,B, *A. callidryas F* = 20.24, *P* < 0.001; *D. ebraccatus F* = 118.78, *P* < 0.001), but not for the metabolites (*A. callidryas* metabolites, *F* = 1.9, *P* = 0.14; *D. ebraccatus F* = 0.05, *P* = 0.99). This pattern again did not seem to correlate linearly with the west-east gradient. The two central sites had higher dispersion than the two extreme sites, which seemed to match the pattern for beta-diversity based on the Adonis tests. This is not surprising, as the multivariate permutational tests can be influenced by both mean tendency and dispersion.

#### Structure-function links and Bd arrival time

There was no correlation between structure (OTUs) and function (metabolites) for either of the treefrog species at any of the four sites (Table 5); therefore we did not pursue examining changes in the relationship from west to east.

**Table 5:** **Results of Mantel tests assessing correlations between bacterial OTU diversity and bacterially-produced metabolite profiles at each of four sites sampled during a field survey assessing the diversity of the bacterial communities on amphibian skin**.

	**Mantel statistic (*r*)**	***P*-value**
***Agalychnis callidryas***		
Campana	0.0484	0.372
Soberania	0.7041	0.335
Mamoni	−0.08357	0.735
NuevoVigia	0.06017	0.344
***Dendropsophus ebraccatus***		
Campana	−0.2941	0.939
Soberania	−0.1401	0.927
Mamoni	0.05204	0.409
NuevoVigia	−0.102	0.585

Results are presented for the two treefrog species, Agalychnis callidryas and Dendropsophus ebraccatus, that were sampled at all four sites. For each species, sites are listed based on the relative length of time these species have persisted with the fungal pathogen, Batrachochytrium dendrobatidis, at each site (i.e., longest at Cerro Campana and shortest at Nuevo Vigia; Woodhams et al., 2008; Rebollar et al., 2014).

## Discussion

We found clear differences in the skin microbiome of the three tropical frog hosts we assessed, including for the two treefrog species that were sampled from the same ponds at the four different sites. The most pronounced differences in relative abundance distributions occurred between the robber frog (*C. fitzingeri*) and the two treefrogs (*A. callidryas* and *D. ebraccatus*). The two treefrogs tended to have more similar relative abundance distributions, suggesting that phylogenetic relatedness and/or habitat overlap may explain some of the variation in skin bacterial community structure. These species-level differences in the amphibian skin microbiota have been documented previously, although primarily from temperate amphibian species (in Colorado, US, McKenzie et al., 2012; in California, US, Kueneman et al., 2014; in Virginia, US, Walke et al., 2014). In tropical systems, differences in the skin microbiota among three *Atelopus* spp. frogs have been seen in Colombia using culture-based methods (Flechas et al., 2012).

These host species differences in the amphibian microbiota are one of the clearest patterns to have emerged from the studies completed to date. The mechanisms driving host species differences are not well understood, although it is likely a combination of differences in environmental reservoirs of bacteria, as well as host and microbe factors that contribute to community assembly of the skin microbiota. In the salamander *Plethodon cinereus*, the structure of the environmental bacterial community appears to be a major determinant of bacterial community structure of the salamanders' skins (Loudon et al., 2014). However, while various members of the amphibian skin microbiota may be found in the environment, environmental differences in the source pool of exposure do not completely explain differences among amphibian host species, as even species inhabiting the same habitats, such as ponds, can have markedly different skin bacterial communities (McKenzie et al., 2012; Walke et al., 2014). Although there has not been extensive work done on species-level differences in the microbiome among other free-living animals, similar species differences have been observed in a few systems, including in marine sponges (Schmitt et al., 2012; Easson and Thacker, 2014), *Hydra* (Franzenburg et al., 2013), and primates (Yildirim et al., 2010).

Only a very small fraction of OTUs were dominant on frog skins despite a large number of total OTUs observed. With this pattern in mind, we can hypothesize that only a small number of OTUs are involved with the host in a stable mutualism, while other OTUs are individually rare and more transient. However, the sum total of the more rare community likely competes with the relatively abundant OTUs, thereby altering the competitive landscape and preventing one OTU from achieving competitive exclusion (Loudon et al., 2014). A focus on the biology of the function of the dominant OTUs is likely to advance our knowledge of how maintaining these symbiotic bacteria might benefit the host.

We also observed host species differences in metabolite diversity, with *A. callidryas* having the most metabolites of the three species we examined. There was also an indication of species differences in the metabolite profiles, but the pattern of separation of the three species was not as clear as it was for the OTU data. The lack of clear species differentiation in metabolite profiles was surprising because in a prior study using the same HPLC-MS methods, Umile et al. (2014) saw very clear differences in the metabolite profiles across 10 amphibian species. Two key distinctions between that study and the present study were that (1) we rinsed frogs to remove dirt and transient microbes prior to collecting the swab samples, which was not done in Umile et al. (2014) and (2) we collected two swab samples, with the second swab always dedicated to the metabolite analyses and the first to the bacterial OTU analysis, while Umile et al. (2014) alternated swab order for bacteria and metabolites. So while we still did see a signal of species level differences in our study, we think that one or both of these factors might have masked that signal relative to the findings of Umile et al. (2014).

In addition to the species level differences in OTU structure within Panamá, we also saw clear separation between the tropical and temperate species in our analysis when comparing the OTUs on the Panamanian frogs with frogs collected in Virginia, US. Indeed, the differences in the relative abundance distributions were obvious for most of the 31 OTUs identified by our K-S approach. At the level of phyla, however, there was broad similarity between Panamá and US frog skin bacteria, with dominance of Proteobacteria and Actinobacteria, and lesser contribution of OTUs in the Bacteroidetes and Firmicutes, although the relative abundances of these groups seemed to shift somewhat across zones. We made no attempt to control for phylogeny in our analysis although we did have Hylids in both groups with *A. callidryas* and *D. ebraccatus* from Panamá and *P. crucifer* from the US, and a future, larger study should do that. Few studies in other systems have investigated temperate vs. tropical host-associated microbes of related host taxa; however, in marine sponges there are distinctions between the microbiota of tropical and sub-tropical species (Schmitt et al., 2012). Our initial finding of differences among tropical and temperate species suggests that a larger study of the biogeography of amphibian skin microbes will be fruitful and might lead to new insights about interactions between Bd and the skin microbiome.

We did not find clear evidence of links between Bd and the amphibian skin microbiota. In terms of individual frogs, for instance, there were no differences in microbiota structure (OTUs) or function (metabolite profiles) based on whether frogs were Bd-positive or Bd-negative at the time of sampling. We did not have a lot of infected frogs in our survey (only 35/136 frogs), and most of these Bd infected frogs had low infection intensities. In particular, with relatively few infected frogs, and those split across three species and four sites, we had low statistical power to detect any possible differences that were based on Bd infection status. In addition, with field sampling we do not know what the individual history of each frog was in relation to Bd. Our samples likely represented a mix of frogs with active infection, some frogs with prior exposure that subsequently cleared infection, and some with no prior exposure, even within Bd endemic sites. In addition, if Bd had already selected for hosts that maintain defensive microbial communities, then we would not expect to see differences in community structure between currently infected and uninfected individuals. Experimental Bd exposure studies have demonstrated that the initial amphibian skin microbiota can influence disease outcome following Bd exposure and that Bd itself can impact the skin bacterial community structure (Harris et al., 2009; Jani and Briggs, 2014; Becker et al., 2015a). We also intentionally focused on three hosts that have not experienced major declines following the arrival of Bd so that we could focus on interactions with the microbiota. The interactions on host species that are more susceptible to Bd infection might be very different than those we observed.

We also saw no clear signal in our data associated with the estimated arrival time of Bd at the various sites across our survey. A lack of long-term data, especially at the eastern-most sites, makes definitive dating of Bd arrival times more difficult in that region. Despite that, we think our hypotheses presented a reasonable expectation of what would have been expected if the wave-like pattern of spread did happen, and we did not find strong support for any of our hypotheses based on that idea. For *A. callidryas*, there did appear to be some increase in mean OTU richness along the west-east gradient, but this was not true for the other treefrog, *D. ebraccatus*. There were also differences in OTU community structure and dispersion across the four sites for the two treefrogs, but the differences were not consistent with a pattern of change occurring from west to east. It is possible that selection on bacterial communities or amphibian hosts is strongest when Bd first arrives and occurs quite rapidly. In that case, we would not expect to see large differences as a function of arrival time of Bd, even though the eastern-most site we sampled likely had Bd present for less than a year when we sampled there. In addition to Bd arrival time, many other factors also vary among the four sites we surveyed, including precipitation patterns, elevation, and even the timing of our sampling within the course of the field season. Any or all of these factors might also impact population level differences at our field sites and warrant further investigation.

While OTU community structure varied at the different sites, we did not see clear clustering of metabolite profiles based on site, and we did not find correlations between OTU and metabolite profile distance matrices. The latter result is interesting in light of current research in ecology focused on structure-function relationships. In “macro”-systems, there is often assumed to be a positive relationship between biodiversity (often estimated by species richness) and ecosystem function. Most tests of this idea though have focused on plant species richness, with primary production as a functional endpoint (Balvanera et al., 2006). In microbial systems, this link between structure and function may be much less pronounced due to the extreme functional redundancy that can occur in systems with thousands of species and the potential for lateral gene transfer. For example, Frossard et al. (2012) found that enzyme activity in soil bacterial communities varied little across environmental gradients despite spatial and temporal variation in bacterial community structure. Our field survey data suggest that multiple different communities of bacteria may be producing the same general sets of metabolites on frog skin, suggesting that there is not a strong link between community structure and function in this system.

## Author contributions

LB, MH, KM, LH, RJ, DM, RI, and RH designed the study, MH, DM, and RI completed fieldwork in Panamá, ER, TU, EB, KM, MB, and JB processed samples in the laboratory, MH, SL, LH, and RJ completed data processing and analysis, LB and MH produced the first draft of the manuscript, and all authors edited the manuscript.

## Funding

This project was funded by the National Science Foundation (DEB-1136640 to LB, LH, and RJ, DEB-1136662 to KM, and DEB-1136602 to RH).

### Conflict of interest statement

The authors declare that the research was conducted in the absence of any commercial or financial relationships that could be construed as a potential conflict of interest.
